# Efficacy of a Guided Web-Based Self-Management Intervention for Depression or Dysthymia: Randomized Controlled Trial With a 12-Month Follow-Up Using an Active Control Condition

**DOI:** 10.2196/15361

**Published:** 2020-07-14

**Authors:** Caroline Oehler, Frauke Görges, Mandy Rogalla, Christine Rummel-Kluge, Ulrich Hegerl

**Affiliations:** 1 German Depression Foundation Leipzig Germany; 2 Department of Psychiatry and Psychotherapy University Leipzig Leipzig Germany; 3 Department of Psychiatry Psychosomatics and Psychotherapy Goethe-Universität Frankfurt Frankfurt Germany

**Keywords:** depression, dysthymic disorder, randomized controlled trial, cognitive behavioral therapy, internet-based intervention, active control, iCBT, self-management, iFightDepression, web-based intervention

## Abstract

**Background:**

An increasing number of studies suggest that web-based interventions for patients with depression can reduce their symptoms and are expected to fill currently existing treatment gaps. However, evidence for their efficacy has mainly been derived from comparisons with wait-list or treatment as usual controls. In particular, designs using wait-list controls are unlikely to induce hope and may even have nocebo effects, making it difficult to draw conclusions about the intervention’s efficacy. Studies using active controls are rare and have not yielded conclusive results.

**Objective:**

The main objective of this study is to assess the acute and long-term antidepressant efficacy of a 6-week, guided, web-based self-management intervention building on the principles of cognitive behavioral therapy (iFightDepression tool) for patients with depression compared with web-based progressive muscle relaxation as an active control condition.

**Methods:**

A total of 348 patients with mild-to-moderate depressive symptoms or dysthymia (according to the Mini International Neuropsychiatric Interview) were recruited online and randomly assigned to 1 of the 2 intervention arms. Acute antidepressant effects after 6 weeks and long-term effects at 3-, 6-, and 12-month follow-up were studied using the Inventory of Depressive Symptomatology–self-rating as a primary outcome parameter and change in quality of life (Short Form 12) and user satisfaction (client satisfaction questionnaire) as secondary outcome parameters. Treatment effects were assessed using mixed model analyses.

**Results:**

Over the entire observation period, a greater reduction in symptoms of depression (*P*=.01) and a greater improvement of life quality (*P*<.001) was found in the intervention group compared with the active control group. Separate tests for each time point revealed significant effects on depressive symptoms at the 3-month follow-up (*d*=0.281; 95% CI 0.069 to 0.493), but not after 6 weeks (*main outcome:* *d*=0.192; 95% CI −0.020 to 0.404) and 6 and 12 months. The intervention was significantly superior to the control condition with respect to user satisfaction (25.31 vs 21.97; t_259_=5.804; *P*<.01).

**Conclusions:**

The fact that antidepressant effects have been found for a guided self-management tool in comparison with an active control strengthens the evidence base for the efficacy of web-based interventions. The antidepressant effect became most prominent at the 3-month follow-up. After 6 weeks of intervention, significant positive effects were observed on life quality but not on depressive symptoms. Although the effect size of such web-based interventions on symptoms of depression might be smaller than that suggested by earlier studies using wait-list control conditions, they can be a cost-effective addition to antidepressants and face-to-face psychotherapy.

**Trial Registration:**

International Clinical Trials Registry Platform ICTRP080-15-09032015; https://apps.who.int/trialsearch/Trial2.aspx?TrialID=DRKS00009323

## Introduction

### Web-Based Interventions in the Treatment of Depression

Web-based interventions for people with depression have been evaluated positively in numerous randomized controlled trials (RCTs). They raise the hope of offering a cost-effective and easily disseminated intervention via the internet [1] for one of the most common disorders worldwide [2]. Cognitive behavioral therapy (CBT) is an evidence-based treatment for depression recommended in national and international treatment guidelines, but access to this treatment is limited [3]. Especially in primary care, where the majority of patients with diagnosed depression are treated [4], access to psychotherapy is problematic. Web-based interventions are promising for closing this treatment.

The majority of web-based interventions for depression are based on techniques derived from CBT, consist of 5 to 15 modules, and incorporate psychoeducational material as well as interactive elements or tasks [5]. Their efficacy seems to have been confirmed by several reviews and meta-analyses finding statistically significant, moderate effect sizes (*d*=0.56 [6] and *d*=0.59 [7]; Hedges *g*=0.50 [8]; *d*=.67 [9]) when comparing internet-based interventions with treatment as usual (TAU) or with wait-list controls. A consistent finding is that interventions that incorporate some kind of guidance (through personal contact or via email support) turn out to have better retention rates and antidepressant effects than self-guided interventions [8,10]. Web-based interventions across several disorders also have been found to produce stable effects for up to 3 years in a review incorporating 14 trials with 902 participants, 3 of which dealt with depression and found positive effects after 2-3.5 years [11].

Although these studies and meta-analyses appear to provide consistent evidence for the efficacy of web-based interventions, an important limitation sheds doubts on this area of research: Effect sizes observed in trials investigating web-based interventions are dependent on the control condition used [12]. Studies with wait-list controls produce larger effect sizes (Hedges *g*=0.9) than those using care as usual or other control conditions (Hedges *g*=0.38) [10]. One possible explanation for this phenomenon is the influence of patients’ expectations regarding the success of the intervention. Especially for patients with depression (a condition in which hopelessness and a negative view of the future are part of the symptomatology), becoming aware of *only* being *in the control condition* does not induce hope but might instead produce nocebo effects [12].

### A Critical Review of the Evidence

So far, most trials on web-based interventions have relied on wait-list and care as usual controls [13], with comparisons to wait-list control likely overestimating the real efficacy. To date, only a few studies have compared web-based interventions for depression with active or placebo control interventions and even fewer report on follow-up data.

Mackinnon et al [14] tested 2 active treatments (a web-based intervention and an information website) against an attention control condition, in which participants discussed certain aspects of their lifestyle with the study team. Although varying in content (depression-specific content vs aspects about lifestyle), all 3 groups received the same amount of telephone contact with the study team [15]. The observed effect sizes for the web-based intervention (*d*=0.38) and the information website (*d*=0.29) were statistically significant but smaller than those in wait-list–controlled studies.

Johansson et al [16] tested a tailored and standardized version of the same web-based intervention against an active control condition (an online discussion forum) and found moderate to large effects with regard to symptoms of depression (*d*=0.84 and *d*=0.57). However, patients randomized into the control arm first received the invitation to join an online discussion forum and, after the intervention period, received the standardized treatment. In this design, patients knew when they were randomized into a control and wait-list condition and might have been disappointed or felt set back by this, potentially leading to less hope induction or even nocebo effects.

Glozier et al [17] conducted a double-blind study on an internet-based cognitive behavioral therapy (iCBT) program. The iCBT intervention was compared with an internet-delivered health intervention for depression in patients with cardiovascular disease and both, participants and study assistants assessing the outcome measure, were unaware of which condition was the active intervention. The iCBT program led to a significantly greater reduction in symptoms of depression, but the effect size in this well-controlled design was small (Cohen *d*=0.16).

Taken together, when using more valid control conditions, the evidence for the antidepressant efficacy of web-based interventions relies on a limited number of studies, and reported effect sizes appear to be small. The best available evidence for antidepressant efficacy thus far stems from a meta-analysis comparing internet-based interventions for depression with face-to-face psychotherapy. Andersson et al [18] combined 5 studies that directly compared guided internet-based interventions with face-to-face psychotherapy (often in a group setting) and found a small effect size in favor of web-based intervention (Hedges *g*=0.12), which was not significantly different from zero.

However, not only the direct intervention effects should be subjected to critical examination. The existing results on long-term efficacy might also be influenced by the choice of controls. Psychotherapeutic interventions are claiming long-term positive effects resulting from the learning of new behavioral and cognitive patterns. Although the first results thus far seem positive, the number of studies incorporating longer follow-up periods is limited and often stems from wait-list–controlled trials. From the 40 high-quality RCTs that were included in a recent meta-analysis for web-based interventions targeting depression [8], only 3 studies contained data on a follow-up of at least 12 months. All 3 studies used within-group comparisons of symptom severity following the intervention to later time points and reported stable effects over the respective follow-up periods [19-21]. This statistical comparison is mainly used in wait-list–controlled trials, which have the disadvantage of not enabling between-group comparisons at follow-up. As symptoms of depression usually fluctuate spontaneously and episodes of depression are usually remitting after several months even when untreated, it is unclear if the results found at follow-up are due to a successful treatment, an initial placebo effect plus spontaneous remission, further treatment options participants took, or other external factors. So far, only a few studies with smaller samples have reported between-group comparisons at follow-up. For example, in one study on 69 participants, using a face-to-face intervention as a comparator, data from a long-term follow-up confirmed that after 3.5 years both groups still did not differ in a statistically significant way [20]. Through the design of this study, it will be possible to expand our understanding of the long-term effects of web-based interventions for depression.

### This Study

The general objective of this study is to strengthen the evidence base for web-based interventions and to close the described gaps in the previous results. To this end, we implemented a web-based active control condition. This control condition was designed to be as similar as possible to the intervention concerning credibility, hope induction, and contact with the study assistants. Furthermore, a 12-month follow-up was implemented.

The main objective was to compare changes in self-rated symptom severity occurring during the trial period up to 12 months for patients with mild-to-moderate depression, who either used a CBT-based, web-based self-management tool (iFightDepression [iFD]) or took part in an active control condition (progressive muscle relaxation [PMR]). We expected the iFD tool to be superior to an active control in terms of symptom reduction. Our main outcome of intervention effects after the 6-week intervention period (as predefined in the study protocol) was supplemented by long-term data to extend the relevance of our conclusions.

Further objectives focused on a more in-depth analysis of the effects of both interventions by doing the following:

- Considering possible covariates such as age, gender, or amount of guidance that might influence the intervention effect.
- Examining the differences between both interventions with respect to changes in self-rated quality of life.
- Examining possible differences between the 2 conditions concerning user experience as well as the amount of usage and the duration and content of guidance in a descriptive and explorative manner.

## Methods

### Trial Design

This study is an RCT assessing the efficacy and usability of a guided web-based self-management intervention (iFD) compared with an active control condition (PMR) after 3 and 6 weeks of intervention as well as after 3, 6, and 12 months postintervention. The trial was conducted in accordance with the Declaration of Helsinki. The complete study protocol was published elsewhere [22] and is in line with the Consolidated Standards of Reporting Trials statement [23].

### Recruitment and Selection of Participants

The study participants were recruited throughout Germany via the website, social media channels, appearances in other media, and newsletters of the German Depression Foundation (DF). Furthermore, newsletters of associated organizations were used for distribution. Interventions were offered free of charge, and no reimbursement was offered to participants.

Individuals interested in taking part in the study were directed to a website providing general information on the study procedures and a web-based questionnaire assessing several inclusion and exclusion criteria. In the study information provided to the participants, both interventions were described as equivalent offers to not induce a bias in expectations. After successfully passing the screening questionnaire, contact details could be left for the main screening that took place via telephone. This procedure led to the preselection of individuals with sufficient internet literacy to meet the minimal study requirements. All screening procedures and guidance during the trial were carried out by psychologists or psychotherapists. If the screening was successful, participants were asked to provide written informed consent for participating in the study and to provide the telephone number of a confidant, whom the study team could contact in case of a suspected crisis (for further details on the screening and inclusion procedures see Oehler et al [22]).

Inclusion criteria were outpatient status, a diagnosis of depressive disorder with presently mild or moderate severity (F32.0, F32.1, F33.0, and F33.1) or dysthymia (F34.1) according to the Mini International Neuropsychiatric Interview (MINI) and patient health questionnaire-9 (PHQ-9; score 5-14, indicating mild-to-moderate symptoms), aged 18 years and above, sufficient language skills to meet the study requirements, and internet access. Outpatient status was taken as one of the inclusion criteria, so that patients could be referred to their local care provider in case of a crisis. Exclusion criteria were dementia, drug or alcohol abuse within the last 6 months, drug or alcohol addiction, schizophrenia, manic episodes or bipolar disorder, obsessive-compulsive disorder (all according to the MINI), known personality disorders (F60.2 and F60.31), acute suicidal tendencies, severe somatic disorders requiring immediate treatment, pregnancy, and participation in another clinical trial within the past 4 weeks.

All participants who provided written informed consent and matched the inclusion and exclusion criteria were randomized using the minimization algorithm by Pocock [24] and stratified for gender (male/female), depression severity (mild/moderate according to PHQ-9), and CBT experience (present/absent) with an 80% chance of using the algorithm’s recommendation.

To the best of our knowledge, no comparable studies were available for power calculation at the time. Therefore, power calculation was based on the results of a study [25], which compared face-to-face CBT with an active control condition (guided self-help group) in depressed primary care patients and found a difference of 5.3 points on the Inventory of Depressive Symptomatology–clinician rated after 10 weeks. For this study, the difference was estimated to be 4.0 points on the Inventory of Depressive Symptomatology–self-rating (IDS-SR), as we expected the difference to be slightly smaller in a web-based trial compared with face-to-face interventions. On the basis of this estimation, 122 patients per group are needed to detect a difference with a power of 80% (α=.05). It was planned to include 360 participants to obtain at least 250 complete data sets after an expected dropout of approximately 30%.

### Ethics and Trial Registration

The protocol for this study was reviewed and approved by the Ethics Committee at the Faculty of Medicine, University of Leipzig, on February 11, 2015.

The trial was registered under the identification code DRKS00009323 at the German Register for Clinical Trials, with the title *Efficacy of an Internet-Based Self-Management Intervention for Adult Primary Care Patients With Mild and Moderate Depression or Dysthymia*. The international registration of the study was carried out via the *International Clinical Trials Registry Platform* with the secondary ID 080-15-09032015.

### Assessments

There were 3 main assessment points during the intervention period (before the intervention and after 3 and 6 weeks of intervention) and 3 follow-up assessments (3, 6, and 12 months after the end of the intervention period). During this period, the primary and secondary outcome measures were collected.

### Measures

#### Primary Outcome

The IDS-SR was used as the primary outcome measure in this study (range 0-84) to assess changes in depression severity. The scale has been shown to be useful in detecting symptom change as well as residual symptoms in patients with depression [26]. The concordant validity with the Beck Depression Inventory and the Hamilton Rating Scale for Depression has been shown to be appropriate (*r*≥.88) [27], and the internal consistency at baseline was acceptable (Cronbach α=.78).

#### Secondary Outcomes

To assess changes in the perceived health-related quality of life, Short-Form 12 (SF-12) was used. It was developed as a practical short form of the Short-Form-36. A mental and a physical component score (both ranging from 0 to 100) can be calculated from the questionnaire answers for which moderate to high convergent validity has been shown in several studies [28,29].

The German version (Fragebogen zur Messung der Patientenzufriedenheit) of the client satisfaction questionnaire-8 (CSQ-8) was used to assess acceptance and feasibility of the interventions. As the questionnaire was originally developed for the evaluation of hospital stays, the wording was slightly adapted to fit web-based interventions. A similar adaptation yielded good internal consistency (omega=.95) [30].

The usage of the intervention was assessed each week during the intervention period using a self-report measure with 2 items. Participants were asked how often they had worked with the intervention during the last week and how much time they had spent on it.

Tracking the objective usage was possible only for the iFD group through the log files of the iFD tool website. Offline use in the form of printed worksheets cannot be tracked. For the PMR group, it was registered if participants downloaded the weekly changing intervention audios. The actual use could not be tracked. Owing to these limitations, both objective measures only served as an approximation and validation of the subjective measures.

#### Monitoring Instrument

To monitor changes and detect possible deteriorations in depressive symptoms over the course of the intervention, the 9-item (PHQ-9; range 0-27) was used. The PHQ-9 is a short, well-validated, and widely used measure [31,32]. The internal consistency at baseline was below the values usually reported for this scale (Cronbach α=.66). During the intervention, patients reporting symptoms indicating severe depression for 3 weeks in a row or acute suicidality were contacted by the study assistants via telephone or email and, if necessary, advised to seek appropriate clinical support. This was necessary in 12 cases. A protocol for managing acute suicidality was established. If patients reported severe symptoms of depression but wanted to continue using the intervention, their cases were discussed with the supervising physician.

#### Adverse Events

At the beginning of the study (T0), after 3 weeks (T1), and after 6 weeks (T2), adverse events were recorded. Every new event that led to the inability to work or that needed medical treatment was recorded, and a possible connection to the intervention was assessed. Events that led to unplanned inpatient treatment or were life-threatening or lethal were classified as serious adverse events. Serious adverse events were passed on to the supervising physician for review.

### Documentation of Guidance

The duration of all planned calls made by the study assistants during the intervention period was recorded and added to provide a sum score for the overall guidance received by each patient. Additionally, the content and perceived quality were rated by the study assistants. Adverse events were recorded and topics relevant to the study, for example, date of next appointment, were discussed. Additional calls to ensure patient safety after they reported suicidal thoughts or severe symptoms of depression in the questionnaires as well as the follow-up calls at T3 to T5 were not counted as guidance.

### Interventions

#### The iFightDepression Tool

The iFD tool is a guided web-based self-management tool based on the principles of CBT. It includes 6 core workshops, each comprising written information, worksheets, exercises, and a mood rating. For this study, participants were asked to use the tool for 6 weeks and to complete 1 workshop per week. Each week’s workshop covered a different topic (eg, an activity diary, monitoring and adapting one’s sleep, or challenging automated negative thoughts). The content and development are described in more detail elsewhere [22,33]. iFD offers the opportunity to complete worksheets on the web or to use a printed version. Patients were asked to try out each workshop and, if helpful, continue using the learned techniques. The iFD tool did not change during the study period, except for a news box on the landing page that was updated approximately once a month.

#### Progressive Muscle Relaxation

In this study, PMR was used as the control condition. During the 6 weeks of intervention, participants were encouraged to practice PMR and to learn how to deliberately induce physical relaxation to reduce stress and mental tension. Lessons range from 13 to 33 min and build on one another, adding more muscle groups every week. At the beginning of each week, participants received a link to download the next lesson. They were instructed to practice on a daily basis, if possible, but at least two or 3 times a week and to integrate the practice into their daily routine.

PMR was chosen as a credible control intervention and is widely used in therapeutic settings, for example, as part of CBT or in the treatment of sleep disorders. The method is also highly accepted by the public as a form of self-help for depression [34,35] and is rated to be helpful for clinically depressed patients (n=736; 38.6%) very or moderately effective and (n=749; 39.2%) slightly effective [36]).

In a systematic review of several relaxation techniques (PMR or similar methods), relaxation was recommended as the first-line treatment in a stepped care approach. Antidepressant effects were visible shortly after relaxation interventions, superior to wait-list and no treatment but inferior to psychotherapy [37], making PMR a suitable choice as a control condition.

### Guidance

Guidance was provided during 5 telephone calls by the study assistants (psychologists and psychotherapists) from the Research Center of the DF, supervised by a senior psychiatrist who was involved in the development of the iFD tool. Comparable with iFD guides outside of the study setting, all study assistants were qualified using the standard web-based seminar and used a guideline for the calls based on the webinar content. The focus of the guidance calls was to motivate the participants rather than discuss the intervention content.

To keep contact with the study staff comparable across both intervention groups, the same guideline was used in the guidance calls for both the iFD and PMR groups, and calls were carried out by the same study assistants.

### Statistical Analysis

To pursue the main objective, changes over time in the primary outcome measure were investigated using a mixed model analysis, which included a random intercept and random slope for each participant. The variance-covariance structure was set to unstructured to avoid any constraints. This approach was adopted to make the best use of incomplete data while minimizing the bias to the parameter estimations [38,39]. All analyses were performed on the intent-to-treat sample using data from all randomized participants. As a sensitivity check, the analysis of the main outcome was repeated for a per-protocol sample (only participants having finished at least four workshops in the iFD tool or downloaded 4 sessions of PMR) and using an imputed data set. The parameter of interest for each model was the time x group interaction, specifying the differential change of symptoms over time attributable to the group assignment. A quadratic term and its interaction with the group variable was added (time x time x group) to allow for parabolic trends over time. Within- and between-group effect sizes (Cohen *d*) were calculated using the difference in means between intervention groups at posttreatment and follow-up for imputed data (with 50 imputations) taking into account the dependence of data collected within participants to avoid the loss of power due to incomplete cases [40] and employing the pooled standard deviation as the standardizer. Confidence intervals of the between-group effect sizes were examined to check for intervention effects at each time point.

Several studies have shown that certain covariates at the participant level, such as adherence to the intervention, participant age, or gender [41-43], can influence dropout rates and intervention success. It is also of interest whether the use of the intervention or the amount of contact with the study team influenced the changes in the outcome measures. Therefore, to meet our second objective, possible covariates (chosen based on the literature on covariates influencing the outcome of interventions for depressed patients) were added stepwise to the mixed model (base model), which contained fixed effects for time, time x time, and a dichotomous variable for the intervention group and their interactions, and were kept in the model if they improved the model fit as measured by the restricted maximum likelihood (REML) criterion at convergence. The following covariates were tested: the amount of guidance received (sum of minutes spent on guidance call per participant), sex (female vs male), age, self-reported amount of time spent working with the intervention (sum score of hours spent on intervention), and self-reported frequency of use (sum of self-reported times worked with the intervention). To control for possible effects of other treatments, a dichotomous variable for *taking antidepressants or receiving psychotherapy at screening* vs *not taking antidepressants or receiving psychotherapy at screening* was added as a possible covariate. Covariates and interactions that did not improve the model fit were excluded from the model.

For the third objective, an equivalent analysis was repeated for quality of life using the SF-12 data.

For all models described, model assumptions were checked using graphical inspections of the plotted residuals and the normal Q-Q plot. The assumptions of normality and homoscedasticity were not violated in any of the models. The inspection of Cook distance estimates yielded several influential data points, but the model results remained unchanged after excluding these. Therefore, in this paper, mixed models using the full sample are reported.

To assess our final objective, descriptive analyses were performed on key features of intervention use and the results of the expectation questionnaire. Group differences in the contents of the guidance calls were tested for significance using a two-sample *Z* test for proportions to compare the frequency of each topic. A two tailed *t* test was performed on the CSQ-8 sum scores (user satisfaction) to assess the group difference statistically.

All analyses were performed using R [44]. For the mixed model calculations, the packages lme4 and lmerTest were used to estimate the model coefficients and corresponding *P* values. Results were calculated using REML estimation and the Kenward-Roger approximation to calculate the denominator degrees of freedom for the performed *t* statistics, as this has been reported to be the most robust way to determine the statistical significance of parameters in mixed models [45].

## Results

### Enrollment and Baseline Characteristics

Recruitment for the trial commenced in June 2016 and was completed in August 2018. Follow-up data were collected until August 2019.

**Figure 1:**
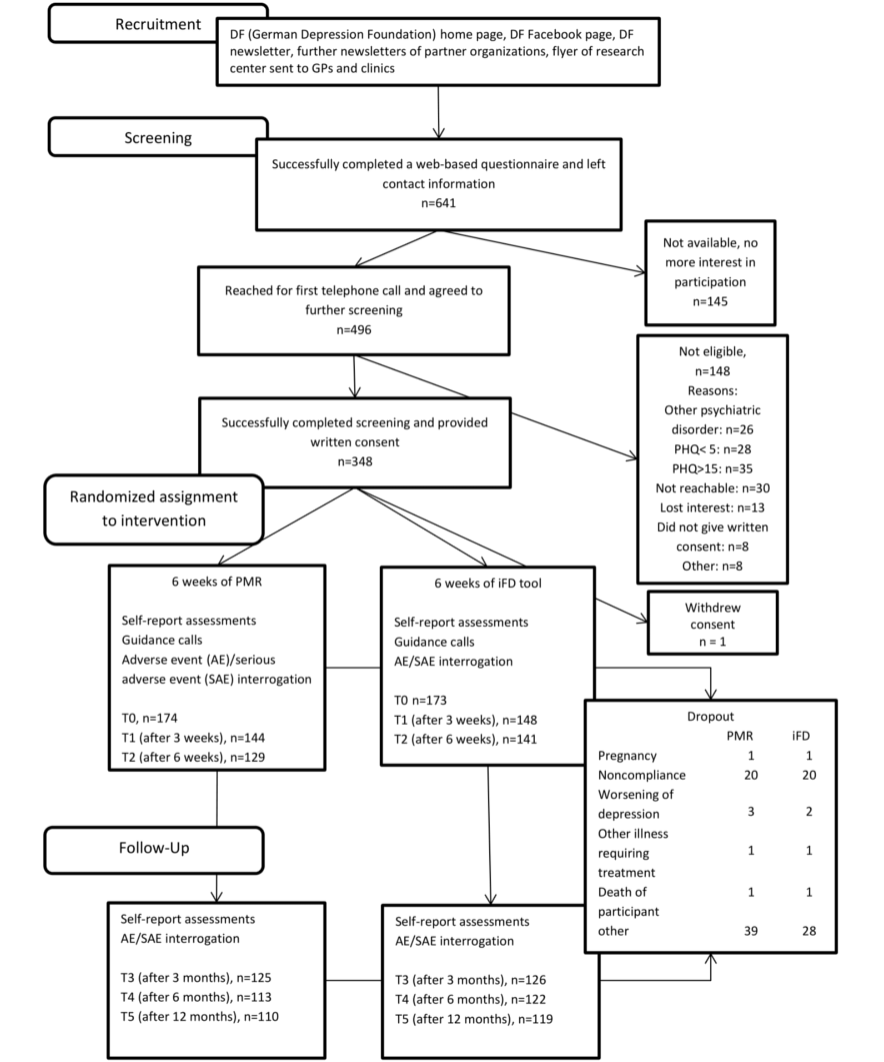
Participant flow chart; a participant was counted as having completed 1 measurement point when either the telephone interview had taken place or the questionnaire had been filled out. Single measures might be reported with slightly differing sample sizes. PHQ: patient health questionnaire; PMR: progressive muscle relaxation; iFD: iFightDepression.

The participant flow chart (Figure 1) provides an overview of the screening and enrollment numbers of the patients (n=347) who were included in the study and randomized into 1 of the 2 treatment arms. The current sample can be described as treatment experienced and, in most cases, with recurrent depression. The levels of both education and internet literacy were high (Table 1).

**Table 1:** Baseline characteristics.

Variable		Intervention group (n=173)		Control group (n=174)	
**Characteristics**					
	Age (years), mean (SD)		42.9 (12.4)		41.7 (12.4)
	Female, n (%)		137 (79.2)		136 (78.3)
	Acquired a university degree, n (%)		64 (37.0)		68 (39.1)
	Internet usage <10 years, n (%)		157 (90.8)		155 (89.1)
	Using the internet daily, n (%)		164 (94.8)		166 (95.4)
**Inclusion diagnosis**					
	Major depression, n (%)		2 (1.2)		4 (2.3)
	Recurrent major depression, n (%)		164 (94.8)		157 (90.2)
	Dysthymia, n (%)		42 (24.3)		41 (23.6)
	Currently under antidepressants, n (%)		115 (66.5)		108 (62.1)
	Currently receiving psychotherapy, n (%)		97 (56.1)		93 (53.1)
	Received psychotherapy in the past, n (%)		169 (97.7)		161 (92.6)
	Psychiatric admission in the past, n (%)		120 (69.4)		117 (67.2)
	Median number of self-reported episodes in the past, n (range)		6 (1-120)		5 (1-150)
**Comorbidities**					
	Social phobia, n (%)		25 (14.5)		23 (13.2)
	Agoraphobia or panic disorder, n (%)		33 (19.1)		27 (15.5)
	Generalized anxiety disorder, n (%)		8 (4.6)		6 (3.4)

### Adherence

Of the 347 patients included, 288 filled out the T1 measure (after 3 weeks) and 262 completed T2 (after 6 weeks). The follow-up measures were completed by 251 participants 3 months after the treatment ended, 235 participants after 6 months, and 229 participants after 12 months. Some of the measures reported have deviating sample sizes (n) due to a small number of participants omitting one or more of the measures.

According to self-report, participants in the intervention group used the iFD tool 23.6 (SD 12.6) times on average and participants in the PMR group practiced 22.4 (SD 9.5) times over the course of the 6-week intervention period. They reported to have spent an average of 6.2 (SD 4.7) hours using the iFD program and an average of 6.5 (SD 5.4) hours using PMR. Neither of these differences reached significance (t_303.26_=0.606, *P*=.55 and t_334.91_=0.620, *P*=.54, respectively).

Objective data, taken from the back end of the iFD tool and the download page of the PMR website, confirmed regular use and downloads of both interventions. The iFD users completed an average of 5.5 (SD 2.1) workshops and spent a mean of 3.8 (SD 3.0) hours using the tool on the web over the course of 18.7 sessions (the end of a session was defined by >30 min of idle time after the last click; time after the last click has not been included in average usage time). PMR users downloaded an average of 4.2 (SD 1.86) of the 6 relaxation lessons (n=165; for 10 participants, downloads had to be enabled differently due to technical problems and were not trackable).

### Main Objective

**Figure 2:**
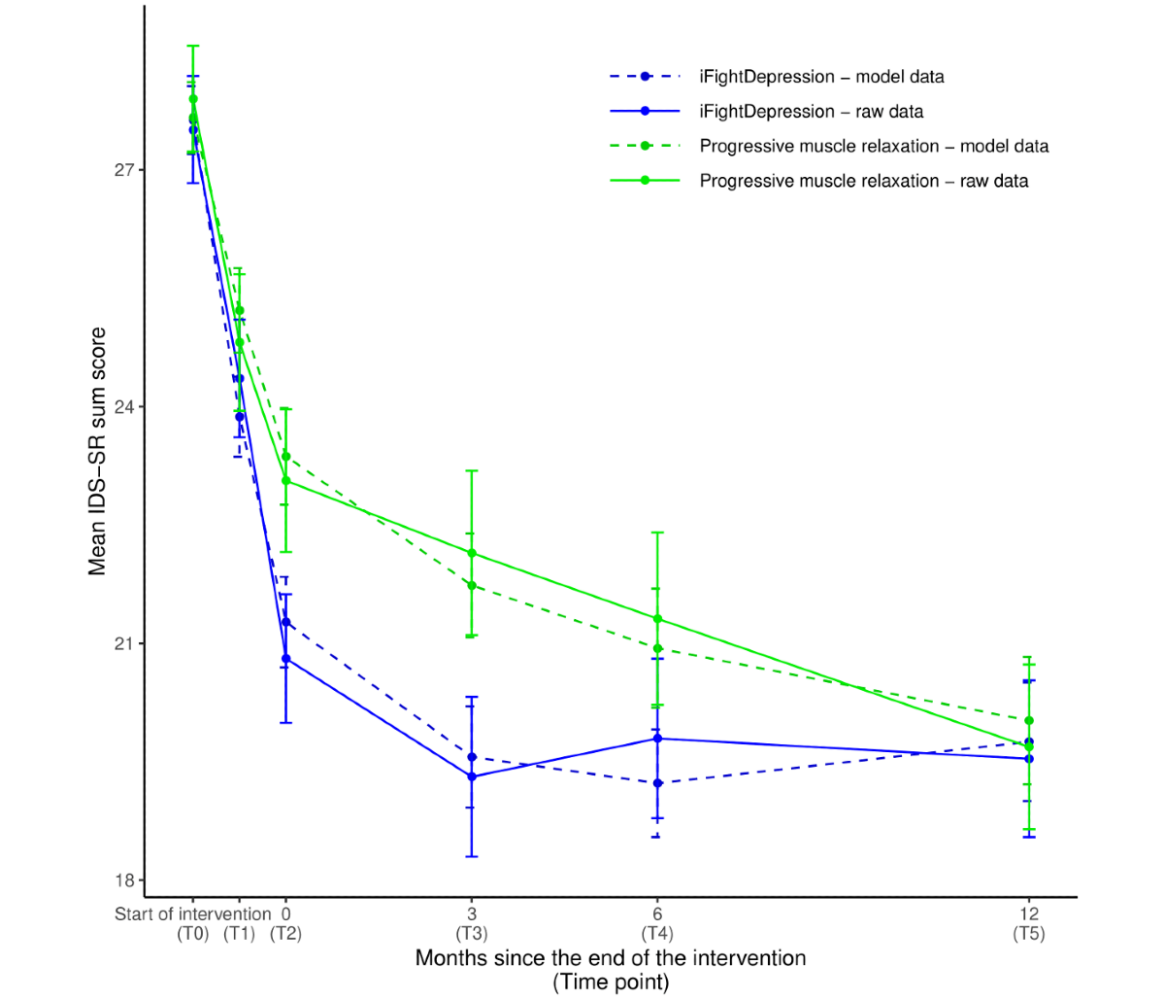
Changes over time in mean IDS-SR. Points of measurement: T0: before the intervention; T1: after 3 weeks of intervention; T2: after intervention period; T3 to T5: follow-up measurements after 3, 6, and 12 months; error bars: standard errors of the mean; and IDS-SR: Inventory of Depressive Symptomatology–self rating. Model results refer to the estimates of the base model including fixed effects for time x group and time x time + group.

Considering intervention effects on the IDS-SR as the primary outcome measure over the entire observation period (6 weeks of intervention + 12 months of follow-up), a significant difference in the symptom change over time was found, favoring the iFD group (an overview of the main and secondary outcomes over time is shown in Table 2). In the base model specified without covariates, the estimated fixed effect of interest (group x time interaction) differed statistically significant from zero (t_1157.2_=−2.519; *P*=.01). A significant main effect of time (t_1196.2_=−3.934; *P*<.001) was also observed, indicating a significant symptom reduction over time in both groups, as well as a significant interaction of time² x group caused by the greater curvature of the trajectory in the iFD group (t_1099.0_=2.686; *P*=.007). Figure 2 depicts the changes in the main outcome variable as well as the values predicted by the base model.

**Table 2:** Mean values for symptoms of depression and quality of life over the course of the intervention, within-group effect sizes.

Intervention		T0 (baseline)		T1 (approximately after 3 weeks)		T2 (approximately after 6 weeks)		T3 (approximately after 3 months)			T4 (approximately after 6 months)			T5 (approximately after 12 months)			Within-group ES^a^ (T0-T2)			Within-group ES^a^ (T2-T5)	
		Mean (SD)	n	Mean (SD)	n	Mean (SD)	n	Mean (SD)	n	Mean (SD)		n	Mean (SD)		n	Cohen *d*		95% CI	Cohen *d*		95% CI
**IDS-SR^b^**																					
	iFD^c^	27.5 (8.9)	173	24.3 (9.1)	148	20.8 (9.4)	135	19.3 (11.4)	126	19.8 (11.2)		122	19.8 (10.8)		119	−0.718		−0.937 to −0.501	−0.182		−0.394 to 0.032
	PMR^d^	27.9 (8.8)	174	24.8 (10.4)	144	23.1 (10.3)	129	22.0 (11.7)	125	21.2 (11.5)		113	19.5 (11.0)		110	−0.619		−0.835 to −0.403	−0.372		−0.584 to −0.159
**SF-12 MCS^e^**																					
	iFD	33.6 (8.3)	173	35.6 (9.0)	146	38.9 (9.7)	133	40.3 (10.8)	126	40.2 (11.5)		122	39.8 (11.1)		119	0.519		0.304 to 0.734	0.056		−0.156 to 0.267
	PMR	33.3 (8.1)	174	34.8 (9.7)	142	36.1 (10.2)	129	37.6 (10.1)	125	38.7 (10.5)		113	41.2 (10.0)		110	0.223		0.012 to 0.435	0.446		0.233 to 0.660
**SF-12 PCS^f^**																					
	iFD	46.9 (9.0)	173	48.1 (9.1)	146	47.3 (9.9)	133	48.8 (9.7)	125	48.7 (8.6)		122	47.8 (9.3)		119	0.093		−0.118 to 0.305	−0.040		−0.252 to 0.171
	PMR	47.2 (9.9)	174	46.8 (9.8)	142	47.3 (9.7)	129	47.9 (8.9)	126	46.4 (9.9)		113	47.0 (9.8)		110	−0.038		−0.249 to 0.173	−0.092		−0.303 to 0.119
**PHQ-9^g^**																					
	iFD	9.1 (3.6)	173	7.9 (3.7)	146	6.9 (3.7)	133	7.0 (4.4)	126	7.4 (4.8)		122	6.7 (4.2)		119	−0.571		−0.898 to −0.356	0.012		−0.120 to 0.223
	PMR	9.7 (3.3)	173	8.2 (3.8)	142	7.4 (3.7)	129	7.9 (4.4)	125	7.9 (4.1)		113	6.7 (4.6)		110	−0.800		−1.019 to −0.580	−0.066		−0.277 to 0.145

^a^ES: effect size, calculated based on imputed data sets.

^b^IDS-SR: Inventory of Depressive Symptomatology–self rating.

^c^iFD: iFightDepression.

^d^PMR: progressive muscle relaxation.

^e^SF-12 MCS: Short-Form 12 mental component score.

^f^SF-12 PCS: Short-Form 12 physical component score.

^g^PHQ-9: patient health questionnaire 9.

As a measure of the model’s ability to describe the data, *R*² was calculated. The base model yielded a conditional *R*² of 0.59 and a marginal *R*² of 0.08, indicating that 59% of the variance in the dependent variable was described by the model and 8% of variance can be explained by the fixed effects alone. The results did not change when using a per-protocol sample or the imputed data set and are therefore not reported separately (results for all models can be found in the Multimedia Appendix 1).

The between-group effect sizes and their confidence intervals, calculated from imputed data sets, are shown in Table 3 and provide an estimate of significance for the group differences at every assessment point. Group differences in the IDS-SR were not statistically significant following the 6-week intervention period (T2) but were so at the 3-month follow-up (T3). Within-group effect sizes comparing T0 and T2 measures (Table 2) can be described as medium according to the rough categorization proposed by Cohen [46] (iFD: *d*=−0.718; PMR: *d*=−0.619). After the intervention period, the IDS-SR scores remained stable in the iFD group and decreased further in the PMR group.

**Table 3:** Between-group effect sizes, results of the mixed models with and without covariates.

Measure	Between-group ES^a^–T2			Between-group ES^a^–T3			Between-group ES^a^–T4			Between-group ES^a^–T5			Fixed effect time x group				Fixed effect time x group		
	Cohen *d*	95% CI	Cohen *d*		95% CI	Cohen *d*		95% CI	Cohen *d*		95% CI	Base model estimate (SE)		*t* value (df)	*P* value	Covariate model estimate (SE)		*t* value (df)	*P* value
IDS-SR^b^	0.192	−0.020 to 0.404	0.281		0.069-0.493	0.030		−0.182 to 0.241	−0.025		−0.236 to 0.186	−2.486 (0.987)		−2.519 (1186.2)	.01	−2.975 (1.098)		−2.710 (941.3)	.007
SF-12 MCS^c^	−0.343	−0.555 to −0.130	−0.249		−0.461 to −0.037	−0.260		−0.472 to −0.048	−0.070		−0.141 to 0.281	3.553 (0.985)		3.608 (1198.4)	<.001	3.531 (1.111)		3.180 (945.7)	*.*002
SF-12 PCS^d^	−0.075	−0.286 to 0.136	−0.205		−0.416 to 0.007	−0.287		−0.499 to −0.075	−0.123		−0.334 to 0.088	0.794 (0.835)		0.950 (1161.1)	.34	0.837 (0.938)		0.892 (932.5)	.37

^a^ES: effect size; between-group effect sizes were corrected for unequal sample size and could therefore also be referred to as Hedges *g*. Effect sizes are calculated based on imputed values to make use of the full data set. Positive values indicate a higher score in the PMR group, and negative values indicate higher scores in the iFD group.

^b^IDS-SR: Inventory of Depressive Symptomatology–self-rating.

^c^SF-12 MCS: Short-Form 12 mental component score.

^d^SF-12 PCS: Short-Form 12 physical component score.

### Secondary Objectives

#### Covariate Analysis

The final model built to predict the IDS-SR scores included the original fixed effects of time, time², and group as well as their interaction as predictors (the full model results are shown in the Multimedia Appendix 1). In addition, fixed effects for self-reported frequency of use, amount of guidance received over the course of the intervention, and the interaction of amount of guidance with the group variable were kept in the model. These covariates were chosen because they improved the model fit (as indicated by a smaller REML criterion at convergence). The significant effect of the amount of guidance (t_216.7_=3.58; *P*<.001) showed that higher overall IDS-SR scores were associated with a greater amount of contact with the study team (model parameter for the fixed effect: 0.20 [SD 0.06]). In addition, the significant interaction of the group and amount of guidance (t_216.4_=−2.13; *P*=.04) mirrors the fact that this was especially the case in the PMR condition.

The fixed effect for frequency of use did not reach statistical significance (t_216.2_=0.58; *P*=.72) but improved the model fit and was therefore kept in the model. The fixed effects of sex, age, taking antidepressants or receiving psychotherapy at screening, and self-reported amount of time spent working with the intervention were not significantly different from zero and did not improve the overall model fit; thus, they were not added to the final model.

None of the other interactions between the covariates and the time or the group variable was significantly different from zero; therefore, the model was specified without additional interactions. This implies that, in this study, the intervention effect was not affected by the covariates named above in a way that was detectable with the current design. The final model yielded a conditional *R*² of 0.60 and a marginal *R*² of 0.10.

#### Quality of Life

**Figure 3:**
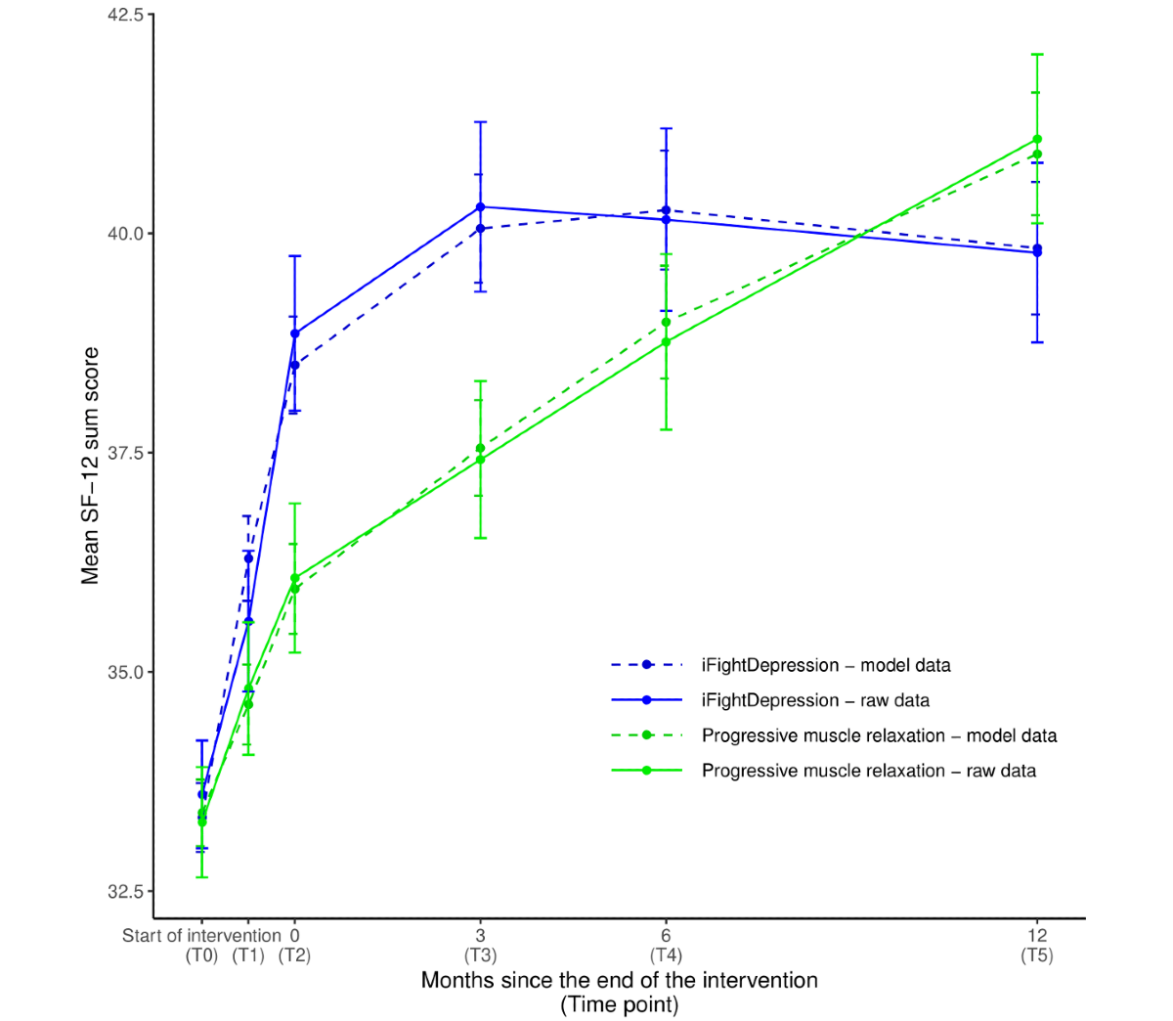
Changes over time in mean SF-12. Points of measurement: T0: before the intervention; T1: after 3 weeks of intervention; T2: after intervention period; T3 to T5: follow-up measurements after 3, 6, and 12 months; error bars: standard errors of the mean; and SF-12: Short-Form 12 (mental component score). Model results refer to the estimates of the base model including fixed effects for time x group and time x time + group.

In the mixed model describing the changes in quality of life (mental component score), the significant interaction of group x time (t_1198.4_=−1.967; *P*=.049) reflects an intervention effect in favor of the iFD tool, indicating a greater improvement in the quality of life for iFD users compared with participants in the PMR group. A statistically significant time² x group interaction indicates greater curvature in the iFD group (t_904.8_=−3.900; *P*<.001). The mental component score was significantly associated with the group variable (t_859.9_=−2.274; *P*=.02; Figure 3), indicating an overall lower score on the SF-12 in the iFD group. The base model yielded a conditional *R*² of 0.55 and a marginal *R*² of 0.06.

The between-group effect sizes after the intervention (T2) and at the 3-month follow-up (T3) showed statistically significant intervention effects (Table 3).

In an extended mixed model containing self-reported frequency of use and the amount of guidance, the covariates themselves did not have a significant effect on the SF-12 scores, but improved the model fit. In addition, the group variable was no longer assigned a significant fixed effect, meaning the model did not yield a significant main effect of intervention group. The final model yielded a conditional *R*² of 0.64 and a marginal *R*² of 0.06.

In the mixed model using the physical component score as a dependent variable, none of the relevant fixed effects significantly differed from zero. As expected, the physical component score was not affected by the intervention and remained unchanged over time (full model results are shown in the Multimedia Appendix 1).

### Guidance

The mean time spent on the 5 guidance and study calls was 38.5 (SD 9.9) min per participant for the iFD group and 28.9 (SD 15.0) min per participant for the PMR group, with the difference being statistically significant (t_314.41_=5.078; *P*<.001). The subjective quality as rated by the guiding study assistants was *positive* or *mostly positive* for 96.7% (736/761) of the calls in the iFD group and for 96.5% (691/716) in the PMR group. The relative occurrence of specific topics during the guidance calls, as recorded by the guides, are reported in Table 4.

**Table 4:** Frequency of specific topics that occurred in the telephone calls, rated by guide, raw values, and percentage (calculated with number of observations/interviews as 100%).

Topic^a^	iFD^b^ (n=761 observations), n (%)	PMR^c^ (n=716 observations), n (%)	Chi-square value (*df*)	*P* value^d^	Holm-corrected *P* value
Comprehension problems	54 (7.0)	4 (0.6)	40.1 (1)	<.001	<.001
Current state of health	658 (86.5)	626 (87.4)	0.2 (1)	.64	.76
Content support	247 (32.5)	126 (17.6)	42.4 (1)	<.001	<.001
Motivation of participant	121 (15.9)	137 (19.1)	2.5 (1)	.12	.59
Dissatisfaction with intervention	83 (10.9)	159 (22.2)	33.6 (1)	<.001	<.001
Positive feedback on the intervention	301 (39.6)	206 (28.8)	1.4 (1)	.23	.76
Questions about released content (only iFD)	27 (3.5)	N/A^e^	N/A	N/A	N/A
Study organizational topics	639 (84.0)	596 (83.2)	0.1 (1)	.76	.76
Irregular participation in intervention	78 (10.3)	61 (8.5)	1.1 (1)	.29	.76

^a^Multiple topics could be the subject of each call; therefore, percentages do not add up to 100.

^b^iFD: iFightDepression.

^c^PMR: progressive muscle relaxation.

^d^*P* values corrected for multiple testing using Hochberg correction.

^e^Not applicable.

#### User Satisfaction

The mean sum score of the CSQ-8, a measure of user satisfaction, was 25.31 in the iFD group and 21.97 in the PMR group out of a possible 32, the difference being statistically significant (t_259_=5.8044; *P*<.01). Satisfaction with the iFD tool seems to be in the expected range, with a sum score similar to those of other studies on internet interventions (26.26 major depression [MD] prevention [30], 26.05 stress management, 24.51 MD treatment in routine psychiatric care [47], 24.96 guided internet-based intervention for depression [48], 22.88 unguided internet-based intervention for depression, and 22.4 happiness training [49]).

#### Adverse Events

During the course of the intervention, 159 adverse events and 4 serious adverse events were recorded. Three adverse events were rated as *possibly related* to the intervention, 2 in the iFD group, and 1 in the PMR group. The *possibly related* adverse events were all deteriorations of the patient’s mood. One patient in each group described the feeling of trying one more intervention that did not help as a contributing factor to the deterioration, and 1 participant in the iFD group reported worse symptoms of depression after reducing her antidepressants without consulting her physician, as she expected to get better by using iFD. Of the serious adverse events, 2 occurred in each intervention group and none were rated as related or possibly related to the intervention. None of the adverse or serious adverse events recorded during the follow-up period were related or possibly related to the intervention.

## Discussion

### Principal Findings

This study is one of the first studies on the antidepressant effects of iCBT using a control condition that is both credible and comparable with the iCBT group in the form of delivery, terms of duration, and contact with the study team. To this end, we assessed between-group differences concerning changes in symptom severity over time as the main objective and possible covariates influencing the intervention effect as well as differences in health-related quality of life as a further objective.

The results concerning our main objective (decrease in symptoms of depression as measured with the IDS-SR) differed significantly between the intervention and control groups according to the mixed model results, favoring the iFD group. This effect was especially apparent at the 3-month follow-up (*d*=0.281, reduction on the IDS-SR was 8.2 points for iFD and 4.8 for PMR), whereas the group difference was only approaching significance after 6 weeks (following the intervention, the main outcome as predefined in the study protocol, *d*=0.192). These effect sizes are considerably smaller than those reported in a meta-analysis for trials using wait-lists as controls (*d*=0.56) but more similar to studies using TAU controls (*d*=0.23) [10]. Finding smaller effects or even no effect, when using an active control condition instead of a wait-list control, is to be expected as placebo effects should affect both groups in a similar manner and nocebo effects should be reduced to a minimum, thereby (mostly) ruling out expectation effects and greater hope induction driving the effect. As studies on depressed patients have been shown to be very susceptible to placebo [50], our finding of significant effects compared with a possible active control that is known to be helpful for depressed patients is noteworthy. The effect sizes are in accordance with the studies by Mackinnon et al [14] and Johansson et al [15], who observed small but significant effects when comparing internet-based interventions to active and attention control conditions.

For further interpretation of the current results, the composition of the sample should be considered. The current sample was a self-selected community sample with a high rate of very educated, internet-affine patients who had experienced several episodes of depression in the past (IFD: median 6; PMR: median 5), who had already gained experience with psychotherapy (past: 330/347, 95.1%; present: 190/347, 54.8%), and who were receiving some kind of treatment at the beginning of the study. The mean age at the onset of depression was 21.8 (SD 12.1) years, indicating that, on average, participants had been experiencing episodes of depression for about 20 years before participating in this study.

This implies that the participants in this study could have already gained a lot of experience in therapeutic techniques and in managing their symptoms, which might have attenuated the treatment effects in comparison to studies treating patients with a first episode of depression or less treatment experience. This might indicate that even experienced patient groups can benefit from this type of intervention. On the other hand, some patients might assume that PMR is less likely to be effective than iCBT and will perceive less hope induction. iFD might be perceived as a new and promising treatment option. This can result in overestimation of the true antidepressant effect.

Patient characteristics such as gender and age as well as the amount of use and guidance did not significantly influence the intervention effect in this study. This is in line with the results on sociodemographic data having no predictive value in an individual patient data meta-analysis by Karyotaki et al [51]. However, Karyotaki et al [51] and Donkin et al [42] reported a positive association between the intervention effect and the amount of intervention received, which could not be replicated in this study. A possible explanation for the lack of this association is that patients might regulate uptake according to their needs, that is, one person might feel that they have received sufficient help after 2 modules, whereas a different person might feel that they need all 6 modules. If this holds true, the total amount of intervention received might not predict the outcome, as it did not in this study. In contrast to previous findings [8,15], the amount of guidance provided in this study did not predict treatment success in the mixed model analysis, possibly due to the fact that the mean amount of guidance (33.7 min) was at the lower end of the 30 to 180 min, which Baumeister et al [52] had previously proposed as an optimal amount of guidance in a review of 14 studies on the impact of guidance in web-based interventions. Larger variation in the amount of guidance, for example, in meta-analysis incorporating several studies with differing designs, might reestablish this effect.

In this study, the 1-year follow-up allowed the analysis of the long-term effects of the interventions. The current results show that although the improvement in the intervention group remained stable, the control condition caught up after 6 months and was not significantly different after 12 months. Our sample had a high proportion of patients with recurrent depressive disorders; therefore, spontaneous remission over the course of the 1-year follow-up is plausible.

For our third objective, assessing changes in quality of life, a statistically significant effect for intervention was found. Although both groups reported improved quality of life concerning their mental health, this increase was significantly more pronounced in the group assigned to the iFD tool with a small but statistically significant between-group effect size after 6 weeks of intervention (*d*=−0.343) and 3 months (*d*=−0.359) postintervention. In their overview of several studies on internet-based interventions for depression, Andrews and Williams [53] reported several trials showing positive effects on both quality of life and disability with moderate to large effect sizes. Our results are in line with this, the smaller magnitude of the effect possibly being caused by the stronger control condition.

To address our fourth objective, this study explored key features of the provision of a web-based intervention with guidance according to a practice-oriented guideline. The average time spent on guidance was 9.6 min longer in the iFD group, and content-related support and comprehension problems were significantly more often part of the guidance calls in the iFD group than in the PMR group. These differences in the amount and content of the guidance calls might reflect a differential need within the groups. Although the PMR training was extended a bit each week, the iFD tool offered new topics and new tasks each week and might have been intellectually more challenging. However, it was also perceived as more positive by the participants, leading to significantly fewer complaints about the intervention during the telephone calls and to a higher score on the satisfaction rating.

In addition to improving treatment outcomes, guidance and contact with the study team are often referred to in the literature as important factors to improve adherence to the intervention. In this study, dropout was acceptable and subjective, and objective measures indicated that usage was high in both groups, with most participants completing the majority of weekly assignments. This implies that this rather small amount of guidance was sufficient, raising hopes that iFD might also prove effective in routine care where guidance will be offered by therapists and physicians. However, the highly structured study environment with an extensive screening interview (not counted as guidance time in this study) and regular questionnaires might have further improved adherence. This should be kept in mind when implementing iFD in routine care.

### Limitations

Several limitations must be considered. First, the active control condition cannot be considered a placebo because there are studies pointing toward an antidepressant effect of PMR [37]. This can lead to an underestimation of the true antidepressant effect of the iCBT intervention. As Hart et al [54] argued, it is almost impossible to construct a psychosocial or behavioral intervention that is a true placebo, that is, an intervention that is harmless, completely inactive or inert, and comparable with the intervention tested. Therefore, we decided to choose an active control that might have affected the outcome measures, but that is thought to be less effective in treating depression while still resembling the web-based intervention in its form and the amount of time patients spend on it. However, this design has the disadvantage of not disentangling spontaneous remission from the effects produced by the control condition.

Second, the amount of guidance was significantly longer in the iFD group despite our best efforts to keep it parallel. Although the amount of guidance was not related to outcome, it cannot be ruled out that this difference had a certain impact on our results.

Third, the generalizability of the results is limited by the fact that the results were obtained within the context of an RCT, which differs from implementation in routine practice. Johansson and Andersson [15] showed that even adding a structured screening interview to otherwise unguided web-based interventions led to increased effects of the intervention, so it is plausible that for the current trial, the study procedures had an impact on the effects reported here. Furthermore, our sample was self-selected and is not representative of all patients with depression. This might have led to more motivated participants, who were more interested in web-based interventions than the average patient. In addition, the trial was conducted by the institution implementing iFD in Germany; thus, the presence of allegiance bias cannot be excluded.

Finally, we did not collect data on additional treatment options that patients might have utilized during the follow-up period. Therefore, we cannot control for possible treatment differences between the iFD and PMR groups during follow-up.

### Conclusions

This study is one of the few studies that used a valid control condition (PMR) as well as a 12-month follow-up. The results confirm that with active controls, the effect sizes are smaller than those in wait-list–controlled designs. Nevertheless, the results strengthen the evidence base for the efficacy of web-based CBT interventions in patients with depressive disorders. Over the entire observation period, the iFD tool was superior to an active control in the reduction of symptoms of depression (*P*=.01) and in the improvement of quality of life (*P*<.001). Although the predefined primary outcome (reduction of symptoms of depression on the IDS-SR after 6 weeks) was not statistically significant, the secondary outcome (quality of life, measured through the mental component score on the SF-12 after 6 weeks) was. Patients in the intervention group reported an accelerated symptom improvement most prominent 3 months after the intervention and, on average, transitioned from moderate to mild symptoms of depression (according to the cutoff values of the IDS-SR [55]). The intervention effects remained stable for up to 12 months, with the control participants continuing to improve and catch up with the intervention group from month 6 onward. A total guidance time of 38.5 min with an acceptable adherence indicates that iFD has the potential to be an efficient complement to treatment and to help reduce treatment gaps in psychosocial interventions. The fact that iFD is free of charge and available in 12 languages makes it an option in many countries worldwide.

## Appendix

Multimedia Appendix 1 Efficacy of a Guided Web-Based Self-Management Intervention for Depression or Dysthymia: Randomized Controlled Trial With a 12-Month Follow-Up Using an Active Control Condition.

Multimedia Appendix 2 CONSORT-eHEALTH checklist (V 1.6.1).

## Abbreviations

CBT: cognitive behavioral therapy
CSQ-8: client satisfaction questionnaire 8
DF: German Depression Foundation
iCBT: internet-based cognitive behavioral therapy
IDS-SR: Inventory of Depressive Symptomatology–self-rating
iFD: iFightDepression
MD: major depression
MINI: Mini International Neuropsychiatric Interview
PHQ-9: patient health questionnaire-9
PMR: progressive muscle relaxation
RCT: randomized controlled trial
REML: restricted maximum likelihood
SF-12: Short Form 12
TAU: treatment as usual
