# Supplementary material for: Efficacy of a Guided Web-Based Self-Management Intervention for Depression or Dysthymia: Randomized Controlled Trial With a 12-Month Follow-Up Using an Active Control Condition
Source: J Med Internet Res. 2020 Jul 14;22(7):e15361. doi: 10.2196/15361 (PMC7388040; doi:10.2196/15361)
Supplement: Multimedia Appendix 1 [file jmir_v22i7e15361_app1.pdf]

Multimedia Appendix - Efficacy of a Guided Web-Based Self-Management Intervention for Depression or Dysthymia: Randomized Controlled Trial With a 12-Month Follow-Up Using an Active Control Condition

SCQ-8

Table 1: per item results of the CSQ-8, with four indicating the most positive evaluation and one the most negative.

Item	Mean iFD (n=135)	Mean PMR (n= 130)	Wilcoxon rank sum test with continuity correction	Holm – corrected *p*-Values
1.How would you rate the quality of the program?	3.13	2.97	W = 10268, p = .002	.006
2. Did the program give you the kind of support you wanted?	3.08	2.62	W = 11372, p<.001	<.001
3. To what extent has the program met your needs?	2.79	2.50	W = 10560, p = .002	.006
4. Would you recommend the program to a friend if he or she needed similar help?	3.48	2.93	W = 12072, p<.001	<.001
5. How satisfied are you with the amount of help you have received here?	3.21	2.95	W = 10126, p<.001	.018
6. Did the program help you deal more appropriately with your problems?	3.15	2.59	W = 12370, p<.001	<.001
7. How satisfied are you with the program on the whole?	3.16	2.7	W = 11644, p<.001	<.001
8. Would you use the program again if you needed help again?	3.32	2.74	W = 11976, p<.001	<.001

IDS-SR

Base Modell

Linear mixed model fit by REML. t-tests use Satterthwaite's method ['lmerModLmerTest']
Formula: value ~ time * Group + I(time^2) * Group + (time | PATNO)
Data: Full data set

REML criterion at convergence: 11498.9

Scaled residuals:

Min	1Q	Median	3Q	Max
-4.2187	-0.5575	-0.0323	0.5253	5.1349

Random effects:

Groups	Name	Variance	Std.Dev.	Corr
PATNO	(Intercept)	46.669	6.831	
	time	1.375	1.173	-0.11
Residual		46.950	6.852	

Number of obs: 1615, groups: PATNO, 347

Fixed effects:

	Estimate	Std. Error	df	t value	Pr(>|t|)
(Intercept)	30.26650	1.11883	1157.23978	27.052	< 2e-16 ***
time	-2.77169	0.70448	1196.16544	-3.934	0.0000882 ***
Group	2.08141	1.57913	1151.22248	1.318	0.18774
I(time^2)	0.16660	0.09987	1115.25168	1.668	0.09556 .
time:Group	-2.48568	0.98676	1186.24928	-2.519	0.01190 *
Group:I(time^2)	0.37466	0.13946	1099.01782	2.686	0.00733 **

Signif. codes: 0 '***' 0.001 '**' 0.01 '*' 0.05 '.' 0.1 ' ' 1

Correlation of Fixed Effects:

	(Intr)	time	Group	I(t^2)	tm:Grp
time	-0.820				
Group	-0.709	0.581			
I(time^2)	0.751	-0.966	-0.532		
time:Group	0.585	-0.714	-0.820	0.689	
Grp:I(tm^2)	-0.538	0.692	0.751	-0.716	-0.966

>

> r.squaredGLMM(IDS.model.q)

R2m	R2c
0.0769583	0.5925718

Base model with per-protocol sample (finished at least 4 Workshops/downloaded 4 PMR lessons)

Linear mixed model fit by REML. t-tests use Satterthwaite's method ['lmerModLmerTest']

Formula: value ~ time * Group + I(time^2) * Group + (time | PATNO)

Data: Completers data set

REML criterion at convergence: 7824.4

Scaled residuals:

Min	1Q	Median	3Q	Max
-3.8761	-0.5591	-0.0696	0.5204	4.9904

Random effects:

Groups	Name	Variance	Std.Dev.	Corr
PATNO	(Intercept)	71.226	8.440	
	time	1.951	1.397	-0.45
Residual	47.340	6.880		

Number of obs: 1091, groups: PATNO, 245

Fixed effects:

	Estimate	Std. Error	df	t value	Pr(>|t|)
(Intercept)	26.05511	2.85142	837.15579	9.138	< 2e-16 ***
time	-0.63332	1.51794	684.34875	-0.417	0.67665
Group	8.02747	3.84189	840.19684	2.089	0.03697 *
I(time^2)	-0.08779	0.18865	659.08694	-0.465	0.64184
time:Group	-5.61598	2.04170	688.77720	-2.751	0.00610 **
Group:I(time^2)	0.74671	0.25349	661.97821	2.946	0.00334 **

Signif. codes: 0 '***' 0.001 '**' 0.01 '*' 0.05 '.' 0.1 ' ' 1

Correlation of Fixed Effects:

	(Intr)	time	Group	I(t^2)	tm:Grp
time	-0.945				
Group	-0.742	0.702			
I(time^2)	0.902	-0.984	-0.669		
time:Group	0.703	-0.743	-0.946	0.732	
Grp:I(tm^2)	-0.671	0.733	0.903	-0.744	-0.984

Base model with imputed data

Linear mixed model fit by REML. t-tests use Satterthwaite's method ['lmerModLmerTest']
Formula: value ~ time * Group + I(time^2) * Group + (time | PATNO)
   Data: Imputed data set

REML criterion at convergence: 14876.1

Scaled residuals:

Min	1Q	Median	3Q	Max
-3.9845	-0.5954	-0.0543	0.5520	4.8244

Random effects:

Groups	Name	Variance	Std.Dev.	Corr
PATNO	(Intercept)	43.0171	6.5587	
	time	0.5985	0.7736	0.08
Residual		52.0705	7.2160	

Number of obs: 2082, groups: PATNO, 347

Fixed effects:

	Estimate	Std. Error	df	t value	Pr(>|t|)
(Intercept)	29.73103	1.09765	1324.94926	27.086	< 2e-16 ***
time	-2.42541	0.64289	1518.07527	-3.773	0.000168 ***
Group[T.1]	2.03139	1.55456	1324.95297	1.307	0.191530
I(time^2)	0.11268	0.08953	1386.00146	1.259	0.208382
time:Group[T.1]	-2.49360	0.91050	1518.07360	-2.739	0.006240 **
Group[T.1]:I(time^2)	0.37111	0.12680	1386.00060	2.927	0.003481 **

Signif. codes: 0 '***' 0.001 '**' 0.01 '*' 0.05 '.' 0.1 ' ' 1

Correlation of Fixed Effects:

	(Intr)	time	Gr[T.1]	I(t^2)	t:G[T.
time	-0.823				
Group[T.1]	-0.706	0.581			
I(time^2)	0.761	-0.975	-0.538		
tm:Grp[T.1]	0.581	-0.706	-0.823	0.688	
G[T.1]:I(^2	-0.538	0.688	0.761	-0.706	-0.975

Full model including all selected covariates

Linear mixed model fit by REML. t-tests use Satterthwaite's method ['lmerModLmerTest']
Formula: value ~ time * Group + I(time^2) * Group + dauer.all * Group +
anz.nutzung + (time | PATNO)
Data: Full data set

REML criterion at convergence: 8978.4

Scaled residuals:

Min	1Q	Median	3Q	Max
-4.2553	-0.5833	-0.0268	0.5352	5.1741

Random effects:

Groups	Name	Variance	Std.Dev.	Corr
PATNO	(Intercept)	44.044	6.637	
	time	1.355	1.164	-0.15
Residual		45.573	6.751	

Number of obs: 1269, groups: PATNO, 222

Fixed effects:

	Estimate	Std. Error	df	t value	Pr(>|t|)
(Intercept)	22.037242	2.513941	316.522038	8.766	< 2e-16 ***
time	-2.210563	0.749064	940.339605	-2.951	0.003245 **
Group	7.457682	3.220897	359.008557	2.315	0.021154 *
I(time^2)	0.098106	0.104840	864.800328	0.936	0.349650
dauer.all	0.198055	0.055357	216.657649	3.578	0.000427 ***
anz.nutzung	0.008388	0.022994	216.270921	0.365	0.715638
time:Group	-2.974658	1.097675	941.310555	-2.710	0.006851 **
Group:I(time^2)	0.430958	0.153534	863.512625	2.807	0.005114 **
Group:dauer.all	-0.147595	0.069400	216.365972	-2.127	0.034575 *

Signif. codes: 0 '***' 0.001 '**' 0.01 '*' 0.05 '.' 0.1 ' ' 1

Correlation of Fixed Effects:

	(Intr)	time	Group	I(t^2)	dar.ll	anz.nt	tm:Grp	G:I(^2
time	-0.414							
Group	-0.645	0.324						
I(time^2)	0.379	-0.967	-0.296					
dauer.all	-0.760	-0.002	0.605	0.000				
anz.nutzung	-0.380	-0.002	-0.060	0.001	-0.034			
time:Group	0.283	-0.682	-0.476	0.660	0.001	0.000		

Grp:I(tm^2)	-0.259	0.660	0.435	-0.683	0.000	0.000	-0.967	
Group:dr.ll	0.598	0.001	-0.811	0.000	-0.798	0.049	-0.001	0.000

> r.squaredGLMM(IDS.model.q4)

R2m	R2c
0.1023094	0.5956643

SF12 – mental component score

Base model

Linear mixed model fit by REML. t-tests use Satterthwaite's method ['lmerModLmerTest']
Formula: value ~ time * Group + I(time^2) * Group + (time | PATNO)
Data: Full data set

REML criterion at convergence: 11366.1

Scaled residuals:

Min	1Q	Median	3Q	Max
-2.8182	-0.6118	-0.0518	0.5837	3.2286

Random effects:

Groups	Name	Variance	Std.Dev.	Corr
PATNO	(Intercept)	42.494	6.519	
	time	1.835	1.354	-0.31
Residual		46.334	6.807	

Number of obs: 1603, groups: PATNO, 346

Fixed effects:

	Estimate	Std. Error	df	t value	Pr(>|t|)
(Intercept)	32.61215	1.10485	1172.48279	29.517	< 2e-16 ***
time	0.66546	0.70263	1207.62066	0.947	0.343778
Group	-3.06440	1.55814	1168.00675	-1.967	0.049455 *
I(time^2)	0.11421	0.09973	1118.29287	1.145	0.252375
time:Group	3.55324	0.98486	1198.38990	3.608	0.000321 ***
Group:I(time^2)	-0.54144	0.13927	1102.15636	-3.888	0.000107 ***

Signif. codes: 0 '***' 0.001 '**' 0.01 '*' 0.05 '.' 0.1 ' ' 1

Correlation of Fixed Effects:

	(Intr)	time	Group	I(t^2)	tm:Grp
time	-0.838				
Group	-0.709	0.594			
I(time^2)	0.755	-0.962	-0.536		
time:Group	0.598	-0.713	-0.838	0.687	
Grp:I(tm^2)	-0.541	0.689	0.756	-0.716	-0.963

> r.squaredGLMM(SF.model.q)

R2m	R2c
0.06428885	0.5475976

Full model including all selected covariates

Linear mixed model fit by REML. t-tests use Satterthwaite's method ['lmerModLmerTest']
Formula: value ~ time * Group + I(time^2) * Group + dauer.all + anz.nutzung + (time | PATNO)
Data: Full data set

REML criterion at convergence: 8983.3

Scaled residuals:

Min	1Q	Median	3Q	Max
-2.8117	-0.6064	-0.0462	0.5816	3.2107

Random effects:

Groups	Name	Variance	Std.Dev.	Corr
PATNO	(Intercept)	47.947	6.924	
	time	1.886	1.373	-0.37
Residual		46.222	6.799	

Number of obs: 1267, groups: PATNO, 222

Fixed effects:

	Estimate	Std. Error	df	t value	Pr(>|t|)
(Intercept)	34.437164	1.979824	428.591283	17.394	< 2e-16 ***
time	0.272576	0.758201	944.910799	0.360	0.719298
Group	-2.344910	1.913250	776.676640	-1.226	0.220715
I(time^2)	0.163336	0.105863	858.954157	1.543	0.123221
dauer.all	-0.023962	0.031988	217.383707	-0.749	0.454601
anz.nutzung	-0.002428	0.022046	217.723747	-0.110	0.912410
time:Group	3.531149	1.110551	945.733376	3.180	0.001523 **
Group:I(time^2)	-0.529251	0.154889	856.811993	-3.417	0.000663 ***

Signif. codes: 0 '***' 0.001 '**' 0.01 '*' 0.05 '.' 0.1 ' ' 1

Correlation of Fixed Effects:

	(Intr)	time	Group	I(t^2)	dar.ll	anz.nt	tm:Grp
time	-0.542						
Group	-0.363	0.563					
I(time^2)	0.485	-0.963	-0.503				
dauer.all	-0.572	-0.001	-0.113	0.000			
anz.nutzung	-0.499	-0.002	-0.032	0.002	0.009		
time:Group	0.370	-0.683	-0.825	0.658	0.002	0.000	
Grp:I(tm^2)	-0.331	0.658	0.738	-0.683	-0.002	0.000	-0.964

>
r.squaredGLMM(SF.model.q1)

R2m	R2c
0.06368429	0.5570092

SF12 – physical component score

Base model

Linear mixed model fit by REML. t-tests use Satterthwaite's method ['lmerModLmerTest']
Formula: value ~ time * Group + I(time^2) * Group + (time | PATNO)
Data: Full data set

REML criterion at convergence: 10976.1

Scaled residuals:

Min	1Q	Median	3Q	Max
-3.5580	-0.5436	0.0960	0.5657	2.9353

Random effects:

Groups	Name	Variance	Std.Dev.	Corr
PATNO	(Intercept)	67.554	8.219	
	time	1.013	1.007	-0.47
Residual		33.232	5.765	

Number of obs: 1603, groups: PATNO, 346

Fixed effects:

	Estimate	Std. Error	df	t value	Pr(>|t|)
(Intercept)	46.77916	1.04449	950.43320	44.787	<2e-16 ***
time	0.34697	0.59639	1168.46857	0.582	0.561
Group	-0.90657	1.47375	945.62285	-0.615	0.539
I(time^2)	-0.05953	0.08475	1088.81903	-0.702	0.483
time:Group	0.79370	0.83543	1161.16700	0.950	0.342
Group:I(time^2)	-0.07321	0.11832	1074.95813	-0.619	0.536

Signif. codes: 0 '***' 0.001 '**' 0.01 '*' 0.05 '.' 0.1 ' ' 1

Correlation of Fixed Effects:

	(Intr)	time	Group	I(t^2)	tm:Grp
time	-0.770				
Group	-0.709	0.546			
I(time^2)	0.678	-0.967	-0.481		
time:Group	0.550	-0.714	-0.770	0.690	
Grp:I(tm^2)	-0.486	0.692	0.678	-0.716	-0.967

Full model including all selected covariates

Linear mixed model fit by REML. t-tests use Satterthwaite's method ['lmerModLmerTest']
Formula: value ~ time * Group + I(time^2) * Group + dauer.all + anz.nutzung + (time | PATNO)
Data: Full data set

REML criterion at convergence: 8650.2

Scaled residuals:

Min	1Q	Median	3Q	Max
-3.5766	-0.5713	0.1061	0.5742	2.9656

Random effects:

Groups	Name	Variance	Std.Dev.	Corr
PATNO	(Intercept)	71.1493	8.4350	
	time	0.9077	0.9527	-0.46
Residual		33.3499	5.7749	

Number of obs: 1267, groups: PATNO, 222

Fixed effects:

	Estimate	Std. Error	df	t value	Pr(>|t|)
(Intercept)	49.53477	2.04638	353.32171	24.206	<2e-16 ***
time	0.53402	0.64061	931.45018	0.834	0.4047
Group	-1.18492	1.82365	598.34750	-0.650	0.5161
I(time^2)	-0.07972	0.08974	856.98147	-0.888	0.3746
dauer.all	-0.07744	0.03489	216.17467	-2.219	0.0275 *
anz.nutzung	-0.02059	0.04814	217.33693	-0.428	0.6694
time:Group	0.83717	0.93824	932.52332	0.892	0.3725
Group:I(time^2)	-0.07504	0.13132	855.41591	-0.571	0.5679

Signif. codes: 0 '***' 0.001 '**' 0.01 '*' 0.05 '.' 0.1 ' ' 1

Correlation of Fixed Effects:

	(Intr)	time	Group	I(t^2)	dar.ll	anz.nt	tm:Grp
time	-0.454						
Group	-0.310	0.512					
I(time^2)	0.398	-0.968	-0.448				
dauer.all	-0.604	-0.001	-0.130	0.000			
anz.nutzung	-0.528	-0.003	-0.037	0.002	0.009		
time:Group	0.310	-0.683	-0.751	0.661	0.003	0.000	
Grp:I(tm^2)	-0.271	0.662	0.658	-0.683	-0.003	0.001	-0.968
