# Supplementary material for: Efficacy of a Guided Web-Based Self-Management Intervention for Depression or Dysthymia: Randomized Controlled Trial With a 12-Month Follow-Up Using an Active Control Condition
Source: J Med Internet Res. 2020 Jul 14;22(7):e15361. doi: 10.2196/15361 (PMC7388040; doi:10.2196/15361)
Supplement: Multimedia Appendix 2 [file jmir_v22i7e15361_app2.pdf]

CONSORT-EHEALTH (V 1.6.1) - Submission/Publication Form

The CONSORT-EHEALTH checklist is intended for authors of randomized trials evaluating web-based and Internet-based applications/interventions, including mobile interventions, electronic games (incl multiplayer games), social media, certain telehealth applications, and other interactive and/or networked electronic applications. Some of the items (e.g. all subitems under item 5 - description of the intervention) may also be applicable for other study designs.

The goal of the CONSORT EHEALTH checklist and guideline is to be
a) a guide for reporting for authors of RCTs,
b) to form a basis for appraisal of an ehealth trial (in terms of validity)

CONSORT-EHEALTH items/subitems are MANDATORY reporting items for studies published in the Journal of Medical Internet Research and other journals / scientific societies endorsing the checklist.

Items numbered 1., 2., 3., 4a., 4b etc are original CONSORT or CONSORT-NPT (non-pharmacologic treatment) items.
Items with Roman numerals (i., ii, iii, iv etc.) are CONSORT-EHEALTH extensions/clarifications.

As the CONSORT-EHEALTH checklist is still considered in a formative stage, we would ask that you also RATE ON A SCALE OF 1-5 how important/useful you feel each item is FOR THE PURPOSE OF THE CHECKLIST and reporting guideline (optional).

Mandatory reporting items are marked with a red *.
In the textboxes, either copy & paste the relevant sections from your manuscript into this form - please include any quotes from your manuscript in QUOTATION MARKS,
or answer directly by providing additional information not in the manuscript, or elaborating on why the item was not relevant for this study.

YOUR ANSWERS WILL BE PUBLISHED AS A SUPPLEMENTARY FILE TO YOUR PUBLICATION IN JMIR AND ARE CONSIDERED PART OF YOUR PUBLICATION (IF ACCEPTED).
Please fill in these questions diligently. Information will not be copyedited, so please use proper spelling and grammar, use correct capitalization, and avoid abbreviations.

DO NOT FORGET TO SAVE AS PDF _AND_ CLICK THE SUBMIT BUTTON SO YOUR ANSWERS ARE IN OUR DATABASE !!!

Citation Suggestion (if you append the pdf as Appendix we suggest to cite this paper in the caption):
Eysenbach G, CONSORT-EHEALTH Group
CONSORT-EHEALTH: Improving and Standardizing Evaluation Reports of Web-based and Mobile Health Interventions
J Med Internet Res 2011;13(4):e126
URL: http://www.jmir.org/2011/4/e126/
doi: 10.2196/jmir.1923
PMID: 22209829

Ihre Antwort ist zu lang. Kürzen Sie einige Sätze in Ihrer Antwort.

Your name *

First Last

Caroline Oehler

Primary Affiliation (short), City, Country *

University of Toronto, Toronto, Canada

German Depression Foundation

Your e-mail address *

abc@gmail.com

caroline.oehler@deutsche-depressionsh

Title of your manuscript *

Provide the (draft) title of your manuscript.

Efficacy of a guided web-based self-management intervention for depression or dysthymia - a randomized controlled trial using an active control condition

Name of your App/Software/Intervention *

If there is a short and a long/alternate name, write the short name first and add the long name in brackets.

iFightDepression

Evaluated Version (if any)

e.g. "V1", "Release 2017-03-01", "Version 2.0.27913"

Release 2016-10

Language(s) *

What language is the intervention/app in? If multiple languages are available, separate by comma (e.g. "English, French")

Ihre Antwort ist zu lang. Kürzen Sie einige Sätze in Ihrer Antwort.

URL of your Intervention Website or App

e.g. a direct link to the mobile app on app in appstore (itunes, Google Play), or URL of the website. If the intervention is a DVD or hardware, you can also link to an Amazon page.

https://tool.ifightdepression.com

URL of an image/screenshot (optional)

Meine Antwort

Accessibility *

Can an enduser access the intervention presently?

- ◯ access is free and open
- ◯ access only for special usergroups, not open
- ◯ access is open to everyone, but requires payment/subscription/in-app purchases
- ◯ app/intervention no longer accessible
- ◉ Sonstiges: the intervention is free to use, guidance by a psychotherapist or

Primary Medical Indication/Disease/Condition *

e.g. "Stress", "Diabetes", or define the target group in brackets after the condition, e.g. "Autism (Parents of children with)", "Alzheimers (Informal Caregivers of)"

Depression

Primary Outcomes measured in trial *

comma-separated list of primary outcomes reported in the trial

Symptoms of depression as measured w

Ihre Antwort ist zu lang. Kürzen Sie einige Sätze in Ihrer Antwort.

Secondary/other outcomes

Are there any other outcomes the intervention is expected to affect?

Changes in the perceived health-related quality of life (measure: Short-Form 12, SF-12), the German version (ZUF-8) of the client satisfaction questionnaire (CSQ-8), descriptives about usage and guidance

Recommended "Dose" *

What do the instructions for users say on how often the app should be used?

- (x) Approximately Daily
- ( ) Approximately Weekly
- ( ) Approximately Monthly
- ( ) Approximately Yearly
- ( ) "as needed"
- ( ) Sonstiges:

Ihre Antwort ist zu lang. Kürzen Sie einige Sätze in Ihrer Antwort.

Approx. Percentage of Users (starters) still using the app as recommended after 3 months *

- [x] unknown / not evaluated
- [ ] 0-10%
- [ ] 11-20%
- [ ] 21-30%
- [ ] 31-40%
- [ ] 41-50%
- [ ] 51-60%
- [ ] 61-70%
- [ ] 71%-80%
- [ ] 81-90%
- [ ] 91-100%
- [ ] Sonstiges:

Ihre Antwort ist zu lang. Kürzen Sie einige Sätze in Ihrer Antwort.

Overall, was the app/intervention effective? *

- ( ) yes: all primary outcomes were significantly better in intervention group vs control
- (•) partly: SOME primary outcomes were significantly better in intervention group vs control
- ( ) no statistically significant difference between control and intervention
- ( ) potentially harmful: control was significantly better than intervention in one or more outcomes
- ( ) inconclusive: more research is needed
- ( ) Sonstiges:

Article Preparation Status/Stage *

At which stage in your article preparation are you currently (at the time you fill in this form)

- ( ) not submitted yet - in early draft status
- (•) not submitted yet - in late draft status, just before submission
- ( ) submitted to a journal but not reviewed yet
- ( ) submitted to a journal and after receiving initial reviewer comments
- ( ) submitted to a journal and accepted, but not published yet
- ( ) published
- ( ) Sonstiges:

Ihre Antwort ist zu lang. Kürzen Sie einige Sätze in Ihrer Antwort.

Journal *

If you already know where you will submit this paper (or if it is already submitted), please provide the journal name (if it is not JMIR, provide the journal name under "other")

- ○ not submitted yet / unclear where I will submit this
- ◉ Journal of Medical Internet Research (JMIR)
- ○ JMIR mHealth and UHealth
- ○ JMIR Serious Games
- ○ JMIR Mental Health
- ○ JMIR Public Health
- ○ JMIR Formative Research
- ○ Other JMIR sister journal
- ○ Sonstiges:

Is this a full powered effectiveness trial or a pilot/feasibility trial? *

- ○ Pilot/feasibility
- ◉ Fully powered

Manuscript tracking number *

If this is a JMIR submission, please provide the manuscript tracking number under "other" (The ms tracking number can be found in the submission acknowledgement email, or when you login as author in JMIR. If the paper is already published in JMIR, then the ms tracking number is the four-digit number at the end of the DOI, to be found at the bottom of each published article in JMIR)

- ◉ no ms number (yet) / not (yet) submitted to / published in JMIR
- ○ Sonstiges:

TITLE AND ABSTRACT

Ihre Antwort ist zu lang. Kürzen Sie einige Sätze in Ihrer Antwort.

1a) TITLE: Identification as a randomized trial in the title

1a) Does your paper address CONSORT item 1a? *

I.e does the title contain the phrase "Randomized Controlled Trial"? (if not, explain the reason under "other")

- (•) yes
- ( ) Sonstiges:

1a-i) Identify the mode of delivery in the title

Identify the mode of delivery. Preferably use “web-based” and/or “mobile” and/or “electronic game” in the title. Avoid ambiguous terms like “online”, “virtual”, “interactive”. Use “Internet-based” only if Intervention includes non-web-based Internet components (e.g. email), use “computer-based” or “electronic” only if offline products are used. Use “virtual” only in the context of “virtual reality” (3-D worlds). Use “online” only in the context of “online support groups”. Complement or substitute product names with broader terms for the class of products (such as “mobile” or “smart phone” instead of “iphone”), especially if the application runs on different platforms.

	1	2	3	4	5	
subitem not at all important	( )	( )	( )	( )	(•)	essential

Does your paper address subitem 1a-i? *

Copy and paste relevant sections from manuscript title (include quotes in quotation marks "like this" to indicate direct quotes from your manuscript), or elaborate on this item by providing additional information not in the ms, or briefly explain why the item is not applicable/relevant for your study

"web-based self-management intervention"

1a-ii) Non-web-based components or important co-interventions in title

Mention non-web-based components or important co-interventions in title, if any (e.g., “with telephone support”).

	1	2	3	4	5	
subitem not at all important	( )	( )	(•)	( )	( )	essential

Ihre Antwort ist zu lang. Kürzen Sie einige Sätze in Ihrer Antwort.

Does your paper address subitem 1a-ii?

Copy and paste relevant sections from manuscript title (include quotes in quotation marks "like this" to indicate direct quotes from your manuscript), or elaborate on this item by providing additional information not in the ms, or briefly explain why the item is not applicable/relevant for your study

"guided"

1a-iii) Primary condition or target group in the title

Mention primary condition or target group in the title, if any (e.g., "for children with Type I Diabetes") Example: A Web-based and Mobile Intervention with Telephone Support for Children with Type I Diabetes: Randomized Controlled Trial

	1	2	3	4	5	
subitem not at all important	○	○	○	○	◉	essential

Does your paper address subitem 1a-iii? *

Copy and paste relevant sections from manuscript title (include quotes in quotation marks "like this" to indicate direct quotes from your manuscript), or elaborate on this item by providing additional information not in the ms, or briefly explain why the item is not applicable/relevant for your study

"...for depression or dysthymia"

1b) ABSTRACT: Structured summary of trial design, methods, results, and conclusions

NPT extension: Description of experimental treatment, comparator, care providers, centers, and blinding status.

1b-i) Key features/functionalities/components of the intervention and comparator in the METHODS section of the ABSTRACT

Mention key features/functionalities/components of the intervention and comparator in the abstract. If possible, also mention theories and principles used for designing the site. Keep in mind the needs of systematic reviewers and indexers by including important synonyms. (Note: Only report in the abstract what the main paper is reporting. If this information is missing from the main body of text, consider adding it)

	1	2	3	4	5	
subitem not at all [illegible]	○	○	○	○	◉	essential

Ihre Antwort ist zu lang. Kürzen Sie einige Sätze in Ihrer Antwort.

Does your paper address subitem 1b-i? *

Copy and paste relevant sections from the manuscript abstract (include quotes in quotation marks "like this" to indicate direct quotes from your manuscript), or elaborate on this item by providing additional information not in the ms, or briefly explain why the item is not applicable/relevant for your study

"...a guided CBT based online self-management intervention (iFightDepression) was tested against an active control condition (progressive muscle relaxation)."

1b-ii) Level of human involvement in the METHODS section of the ABSTRACT

Clarify the level of human involvement in the abstract, e.g., use phrases like “fully automated” vs. “therapist/nurse/care provider/physician-assisted” (mention number and expertise of providers involved, if any). (Note: Only report in the abstract what the main paper is reporting. If this information is missing from the main body of text, consider adding it)

	1	2	3	4	5	
subitem not at all important	○	○	○	◉	○	essential

Does your paper address subitem 1b-ii?

Copy and paste relevant sections from the manuscript abstract (include quotes in quotation marks "like this" to indicate direct quotes from your manuscript), or elaborate on this item by providing additional information not in the ms, or briefly explain why the item is not applicable/relevant for your study

"...a guided..." The manuscript itself provides more information on how and by whom guidance was provided, more details would have overburdened the abstract.

Ihre Antwort ist zu lang. Kürzen Sie einige Sätze in Ihrer Antwort.

1b-iii) Open vs. closed, web-based (self-assessment) vs. face-to-face assessments in the METHODS section of the ABSTRACT

Mention how participants were recruited (online vs. offline), e.g., from an open access website or from a clinic or a closed online user group (closed usergroup trial), and clarify if this was a purely web-based trial, or there were face-to-face components (as part of the intervention or for assessment). Clearly say if outcomes were self-assessed through questionnaires (as common in web-based trials). Note: In traditional offline trials, an open trial (open-label trial) is a type of clinical trial in which both the researchers and participants know which treatment is being administered. To avoid confusion, use “blinded” or “unblinded” to indicated the level of blinding instead of “open”, as “open” in web-based trials usually refers to “open access” (i.e. participants can self-enrol). (Note: Only report in the abstract what the main paper is reporting. If this information is missing from the main body of text, consider adding it)

	1	2	3	4	5	
subitem not at all important	○	○	○	◉	○	essential

Does your paper address subitem 1b-iii?

Copy and paste relevant sections from the manuscript abstract (include quotes in quotation marks "like this" to indicate direct quotes from your manuscript), or elaborate on this item by providing additional information not in the ms, or briefly explain why the item is not applicable/relevant for your study

"A total of 348 patients with mild to moderate depressive symptoms or dysthymia were recruited online and randomly assigned to one of the two six-week intervention arms. Symptoms of depression (IDS-SR) as well as quality of life (SF12) and user satisfaction (CSQ-8) were measured in self-report before and after three and six weeks of intervention."

1b-iv) RESULTS section in abstract must contain use data

Report number of participants enrolled/assessed in each group, the use/uptake of the intervention (e.g., attrition/adherence metrics, use over time, number of logins etc.), in addition to primary/secondary outcomes. (Note: Only report in the abstract what the main paper is reporting. If this information is missing from the main body of text, consider adding it)

	1	2	3	4	5	
subitem not at all important	○	○	○	◉	○	essential

Ihre Antwort ist zu lang. Kürzen Sie einige Sätze in Ihrer Antwort.

Does your paper address subitem 1b-iv?

Copy and paste relevant sections from the manuscript abstract (include quotes in quotation marks "like this" to indicate direct quotes from your manuscript), or elaborate on this item by providing additional information not in the ms, or briefly explain why the item is not applicable/relevant for your study

"The usage of both interventions was sufficiently similar (iFightDepression: 6.2 hours, control: 6.5 hours spent on the intervention on average), and the average time spent on guidance was acceptable (iFightDepression: 38.5 min, control: 28.9 min)."

1b-v) CONCLUSIONS/DISCUSSION in abstract for negative trials

Conclusions/Discussions in abstract for negative trials: Discuss the primary outcome - if the trial is negative (primary outcome not changed), and the intervention was not used, discuss whether negative results are attributable to lack of uptake and discuss reasons. (Note: Only report in the abstract what the main paper is reporting. If this information is missing from the main body of text, consider adding it)

	1	2	3	4	5	
subitem not at all important	○	○	○	○	◉	essential

Does your paper address subitem 1b-v?

Copy and paste relevant sections from the manuscript abstract (include quotes in quotation marks "like this" to indicate direct quotes from your manuscript), or elaborate on this item by providing additional information not in the ms, or briefly explain why the item is not applicable/relevant for your study

"Both interventions were associated with a reduction in symptoms of depression. The effects on quality of life as well as user satisfaction ratings were superior for the online self-management intervention. Our results implicate that the effect of web-based interventions on symptoms of depression might be smaller than suggested by earlier studies using wait list or TAU control conditions. However, since self-management tools are not a replacement for antidepressants or face-to-face therapy, even small effects can be cost effective."

INTRODUCTION

2a) In INTRODUCTION: Scientific background and explanation of rationale

Ihre Antwort ist zu lang. Kürzen Sie einige Sätze in Ihrer Antwort.

2a-i) Problem and the type of system/solution

Describe the problem and the type of system/solution that is object of the study: intended as stand-alone intervention vs. incorporated in broader health care program? Intended for a particular patient population? Goals of the intervention, e.g., being more cost-effective to other interventions, replace or complement other solutions? (Note: Details about the intervention are provided in "Methods" under 5)

	1	2	3	4	5	
subitem not at all important	○	○	○	◉	○	essential

Does your paper address subitem 2a-i? *

Copy and paste relevant sections from the manuscript (include quotes in quotation marks "like this" to indicate direct quotes from your manuscript), or elaborate on this item by providing additional information not in the ms, or briefly explain why the item is not applicable/relevant for your study

The current paper is trying to expand the knowledge about efficacy of web-based interventions using an active control condition. Therefore the focus is, on the one hand, to improve the treatment of depression, but on the other hand, to reevaluate efficacy in taking into account possible placebo effects.
"Nevertheless, it is important that new trials on web-based interventions for depression move on to other control conditions to further improve the evidence base and put the results of past trials into perspective.
The objective of the current study is to strengthen the evidence base for web-based interventions and to close the described gaps in the previous results. To this end, we implemented an active control condition, designed to be as similar as possible to the intervention in credibility, hope induction and contact to the study assistants as comparator to an web-based intervention for depression."

2a-ii) Scientific background, rationale: What is known about the (type of) system

Scientific background, rationale: What is known about the (type of) system that is the object of the study (be sure to discuss the use of similar systems for other conditions/diagnoses, if appropiate), motivation for the study, i.e. what are the reasons for and what is the context for this specific study, from which stakeholder viewpoint is the study performed, potential impact of findings [2]. Briefly justify the choice of the comparator.

	1	2	3	4	5	
subitem not at all important	○	○	○	○	◉	essential

Ihre Antwort ist zu lang. Kürzen Sie einige Sätze in Ihrer Antwort.

Does your paper address subitem 2a-ii? *

Copy and paste relevant sections from the manuscript (include quotes in quotation marks "like this" to indicate direct quotes from your manuscript), or elaborate on this item by providing additional information not in the ms, or briefly explain why the item is not applicable/relevant for your study

"Web-based interventions for people suffering from depression have been evaluated positively in numerous randomized controlled trials (RCTs). They raise the hope of offering a cost-effective and easily disseminated intervention via the Internet (Paganini et al. 2018) for one of the most common diseases worldwide (Vos et al. 2016). " [...] "Their efficacy seems to have been confirmed by several reviews and meta-analyses finding statistically significant, moderate effect sizes (d = 0.56; Richards und Richardson 2012 and d = 0.59; Twomey et al. 2015, g = 0.50; Wright et al. 2019, d=.67 Andrews et al. 2018) when comparing internet baes interventions to treatment as usual (TAU) or to wait list controls (WL). A consistent finding is that interventions that incorporate some kind of guidance (through personal contact or via email support) turnout to have better retention rates and antidepressant effects than self-guided interventions (Andersson und Cuijpers 2009), Wright et al. 2019." [...] "So far, only a number of studies investigated internet based intervention for depression in comparison to active or placebo control interventions." [...] "So, on a closer examination, the evidence in favor of web-based interventions obtained so far might be overestimated by the designs that were applied and, thus, should be critically inspected.
The best available evidence so far stems from a meta-analysis comparing internet based interventions for depression to face-to-face psychotherapy. Andersson et al. (2016) combined five studies that directly compared guided internet based interventions to face-to-face psychotherapy (often in a group setting) and found a small effect size in favor of internet based intervention (Hedge’s g =0.12), that was not significantly different from zero. The fact that there was no relevant numerical difference in antidepressant effects suggests that the resulting non-significant difference is not explained by a lack of statistical power."

2b) In INTRODUCTION: Specific objectives or hypotheses

Ihre Antwort ist zu lang. Kürzen Sie einige Sätze in Ihrer Antwort.

Does your paper address CONSORT subitem 2b? *

Copy and paste relevant sections from the manuscript (include quotes in quotation marks "like this" to indicate direct quotes from your manuscript), or elaborate on this item by providing additional information not in the ms, or briefly explain why the item is not applicable/relevant for your study

"The primary objective was to:
1) compare changes in self-rated symptom severity occurring during the six weeks intervention period for patients suffering from mild- to moderate depression, who either used a CBT based online self-management tool or took part in an active control condition. The hypothesis is, that a web-based intervention is superior to an active control in terms of symptom reduction.

Secondary objectives focused on further exploring the effects of both interventions through:
2) adding possible covariates that might influence the primary outcome. Based on the literature it is likely, that certain covariates could influence the effectiveness of online interventions.
3) examining differences between both conditions with respect to changes in self rated quality of life. It is hypothesized, that the web-based intervention leads to a greater improvement in quality of life.
4) examining differences between both conditions concerning user experience and as well as the amount of usage and guidance in an explorative way. "

METHODS

3a) Description of trial design (such as parallel, factorial) including allocation ratio

Ihre Antwort ist zu lang. Kürzen Sie einige Sätze in Ihrer Antwort.

Does your paper address CONSORT subitem 3a? *

Copy and paste relevant sections from the manuscript (include quotes in quotation marks "like this" to indicate direct quotes from your manuscript), or elaborate on this item by providing additional information not in the ms, or briefly explain why the item is not applicable/relevant for your study

"The current study is a RCT comparing the efficacy and usability of a guided online-self-management intervention (iFightDepression®) with an active control condition (progressive muscle relaxation, short: PMR-training) after six weeks of intervention." [...] "All participants, who provided written informed consent and matched the in- and exclusion criteria were randomized using the minimization algorithm by Pocock [29], stratified for gender (male/female), depression severity (mild/moderate according to PHQ-9), and CBT-experience (present/absent) with an 80% chance of using the algorithm's recommendation."

3b) Important changes to methods after trial commencement (such as eligibility criteria), with reasons

Does your paper address CONSORT subitem 3b? *

Copy and paste relevant sections from the manuscript (include quotes in quotation marks "like this" to indicate direct quotes from your manuscript), or elaborate on this item by providing additional information not in the ms, or briefly explain why the item is not applicable/relevant for your study

No important changes to the trial procedures have been made.

3b-i) Bug fixes, Downtimes, Content Changes

Bug fixes, Downtimes, Content Changes: ehealth systems are often dynamic systems. A description of changes to methods therefore also includes important changes made on the intervention or comparator during the trial (e.g., major bug fixes or changes in the functionality or content) (5-iii) and other “unexpected events” that may have influenced study design such as staff changes, system failures/downtimes, etc. [2].

	1	2	3	4	5	
subitem not at all important	○	○	◉	○	○	essential

Does your paper address subitem 3b-i?

Copy and paste relevant sections from the manuscript (include quotes in quotation marks "like this" to indicate direct quotes from your manuscript), or elaborate on this item by providing additional information not in the ms, or briefly explain why the item is not applicable/relevant for your study

There were no major changes or fixes to the intervention during the study

Ihre Antwort ist zu lang. Kürzen Sie einige Sätze in Ihrer Antwort.

4a) Eligibility criteria for participants

Does your paper address CONSORT subitem 4a? *

Copy and paste relevant sections from the manuscript (include quotes in quotation marks "like this" to indicate direct quotes from your manuscript), or elaborate on this item by providing additional information not in the ms, or briefly explain why the item is not applicable/relevant for your study

"Inclusion criteria were: Outpatient status, a diagnosis of depressive disorder with presently mild or moderate severity (F 32.0, F32.1, F33.0, F33.1) or dysthymia (F34.1) according to the M.I.N.I. and the PHQ-9 (score 5-14, indicating mild to moderate symptoms), age ≥ 18 years, sufficient language skills to meet the study requirements and internet access. Exclusion criteria were: Dementia, drug/alcohol abuse within the last 6 months, drug/alcohol addiction, schizophrenia, manic episodes or bipolar disorder, obsessive-compulsive disorder (all according to the M.I.N.I.), known personality disorders (F60.2, F60.31), acute suicidal tendencies, severe somatic disorders requiring immediate treatment, pregnancy and participation in another clinical trial within the past four weeks."

4a-i) Computer / Internet literacy

Computer / Internet literacy is often an implicit “de facto” eligibility criterion - this should be explicitly clarified.

	1	2	3	4	5	
subitem not at all important	○	○	◉	○	○	essential

Does your paper address subitem 4a-i?

Copy and paste relevant sections from the manuscript (include quotes in quotation marks "like this" to indicate direct quotes from your manuscript), or elaborate on this item by providing additional information not in the ms, or briefly explain why the item is not applicable/relevant for your study

"This procedure leads to a preselection of individuals with sufficient internet literacy to meet the minimal study requirements."

Ihre Antwort ist zu lang. Kürzen Sie einige Sätze in Ihrer Antwort.

4a-ii) Open vs. closed, web-based vs. face-to-face assessments:

Open vs. closed, web-based vs. face-to-face assessments: Mention how participants were recruited (online vs. offline), e.g., from an open access website or from a clinic, and clarify if this was a purely web-based trial, or there were face-to-face components (as part of the intervention or for assessment), i.e., to what degree got the study team to know the participant. In online-only trials, clarify if participants were quasi-anonymous and whether having multiple identities was possible or whether technical or logistical measures (e.g., cookies, email confirmation, phone calls) were used to detect/prevent these.

	1	2	3	4	5	
subitem not at all important	○	○	◉	○	○	essential

Does your paper address subitem 4a-ii? *

Copy and paste relevant sections from the manuscript (include quotes in quotation marks "like this" to indicate direct quotes from your manuscript), or elaborate on this item by providing additional information not in the ms, or briefly explain why the item is not applicable/relevant for your study

"The study participants were recruited throughout Germany via the website, social media channels, appearances in other media and newsletter of the German Depression Foundation (DF). Furthermore, newsletters of associated organizations were used for distribution.
Individuals interested in taking part in the study were directed to a website providing general information on the study procedures and an online questionnaire assessing several in- and exclusion criteria. After successfully passing the questionnaire, contact details could be left for the main screening that took place via telephone. This procedure leads to a preselection of individuals with sufficient internet literacy to meet the minimal study requirements. All screening procedures and the guidance during the trial were carried out by psychologists or psychotherapists. If the screening was successful, participants were asked to provide written informed consent for participating in the study and to provide the telephone number of a confidant, whom the study team could contact in a suspected crisis (for further details on the screening and inclusion procedures see Oehler et al. 2019)."

Ihre Antwort ist zu lang. Kürzen Sie einige Sätze in Ihrer Antwort.

4a-iii) Information giving during recruitment

Information given during recruitment. Specify how participants were briefed for recruitment and in the informed consent procedures (e.g., publish the informed consent documentation as appendix, see also item X26), as this information may have an effect on user self-selection, user expectation and may also bias results.

	1	2	3	4	5	
subitem not at all important	○	○	○	◉	○	essential

Does your paper address subitem 4a-iii?

Copy and paste relevant sections from the manuscript (include quotes in quotation marks "like this" to indicate direct quotes from your manuscript), or elaborate on this item by providing additional information not in the ms, or briefly explain why the item is not applicable/relevant for your study

The study information was provided in written form in german language and during the first phone call and is therefore not added as an appedix. The Participants were comprehensively informed about the study procedures, the assessment points and the two possible interventions. They had to be willing to participate in either of the interventions (iFightDepresssion or progressive muscle relaxation). "In the study information provided to the participants, both interventions were described as equivalent offers to not induce a bias in the expectations. "

4b) Settings and locations where the data were collected

Does your paper address CONSORT subitem 4b? *

Copy and paste relevant sections from the manuscript (include quotes in quotation marks "like this" to indicate direct quotes from your manuscript), or elaborate on this item by providing additional information not in the ms, or briefly explain why the item is not applicable/relevant for your study

"During the six-week intervention period, the participants were asked to complete online questionnaires weekly (for details see figure 2)"

4b-i) Report if outcomes were (self-)assessed through online questionnaires

Clearly report if outcomes were (self-)assessed through online questionnaires (as common in web-based trials) or otherwise.

	1	2	3	4	5
subitem not at all	○	○	◉	○	○

Ihre Antwort ist zu lang. Kürzen Sie einige Sätze in Ihrer Antwort.

Does your paper address subitem 4b-i? *

Copy and paste relevant sections from the manuscript (include quotes in quotation marks "like this" to indicate direct quotes from your manuscript), or elaborate on this item by providing additional information not in the ms, or briefly explain why the item is not applicable/relevant for your study

"During the six-week intervention period, the participants were asked to complete online questionnaires weekly (for details see figure 2)"

4b-ii) Report how institutional affiliations are displayed

Report how institutional affiliations are displayed to potential participants [on ehealth media], as affiliations with prestigious hospitals or universities may affect volunteer rates, use, and reactions with regards to an intervention.(Not a required item – describe only if this may bias results)

	1	2	3	4	5	
subitem not at all important	○	◉	○	○	○	essential

Does your paper address subitem 4b-ii?

Copy and paste relevant sections from the manuscript (include quotes in quotation marks "like this" to indicate direct quotes from your manuscript), or elaborate on this item by providing additional information not in the ms, or briefly explain why the item is not applicable/relevant for your study

The German Depression Foundation is mentioned in the study information as the organisation conducting the experiment, but this is not described in detail in the manuscript, as this applies to both intervention arms and we do not expect it to lead to bias.

5) The interventions for each group with sufficient details to allow replication, including how and when they were actually administered

5-i) Mention names, credential, affiliations of the developers, sponsors, and owners

Mention names, credential, affiliations of the developers, sponsors, and owners [6] (if authors/evaluators are owners or developer of the software, this needs to be declared in a "Conflict of interest" section or mentioned elsewhere in the manuscript).

	1	2	3	4	5	
subitem not at all important	○	○	○	◉	○	essential

Ihre Antwort ist zu lang. Kürzen Sie einige Sätze in Ihrer Antwort.

Does your paper address subitem 5-i?

Copy and paste relevant sections from the manuscript (include quotes in quotation marks "like this" to indicate direct quotes from your manuscript), or elaborate on this item by providing additional information not in the ms, or briefly explain why the item is not applicable/relevant for your study

"The content and development is described in more details elsewhere (Oehler et al. 2019, Arensman et al. 2015)." [...] UH, CRK, MR, FG and CO work or have worked for the German Depression Foundation, implementing the iFightDepression tool in Germany.

5-ii) Describe the history/development process

Describe the history/development process of the application and previous formative evaluations (e.g., focus groups, usability testing), as these will have an impact on adoption/use rates and help with interpreting results.

	1	2	3	4	5	
subitem not at all important	○	○	●	○	○	essential

Does your paper address subitem 5-ii?

Copy and paste relevant sections from the manuscript (include quotes in quotation marks "like this" to indicate direct quotes from your manuscript), or elaborate on this item by providing additional information not in the ms, or briefly explain why the item is not applicable/relevant for your study

This information has been provided in other papers citet in the current one (See 5-i).

5-iii) Revisions and updating

Revisions and updating. Clearly mention the date and/or version number of the application/intervention (and comparator, if applicable) evaluated, or describe whether the intervention underwent major changes during the evaluation process, or whether the development and/or content was “frozen” during the trial. Describe dynamic components such as news feeds or changing content which may have an impact on the replicability of the intervention (for unexpected events see item 3b).

	1	2	3	4	5	
subitem not at all important	○	○	●	○	○	essential

Ihre Antwort ist zu lang. Kürzen Sie einige Sätze in Ihrer Antwort.

Does your paper address subitem 5-iii?

Copy and paste relevant sections from the manuscript (include quotes in quotation marks "like this" to indicate direct quotes from your manuscript), or elaborate on this item by providing additional information not in the ms, or briefly explain why the item is not applicable/relevant for your study

The intervention was not changed during the study period, except for a news box on the landing page that was updated approximately once a month.

5-iv) Quality assurance methods

Provide information on quality assurance methods to ensure accuracy and quality of information provided [1], if applicable.

	1	2	3	4	5	
subitem not at all important	○	○	○	◉	○	essential

Does your paper address subitem 5-iv?

Copy and paste relevant sections from the manuscript (include quotes in quotation marks "like this" to indicate direct quotes from your manuscript), or elaborate on this item by providing additional information not in the ms, or briefly explain why the item is not applicable/relevant for your study

The current manuscript does not provide in depth information on quality assurance, but the published study protocol does. Please see Oehler et al. (2019) for further details.

5-v) Ensure replicability by publishing the source code, and/or providing screenshots/screen-capture video, and/or providing flowcharts of the algorithms used

Ensure replicability by publishing the source code, and/or providing screenshots/screen-capture video, and/or providing flowcharts of the algorithms used. Replicability (i.e., other researchers should in principle be able to replicate the study) is a hallmark of scientific reporting.

	1	2	3	4	5	
subitem not at all important	○	○	◉	○	○	essential

Ihre Antwort ist zu lang. Kürzen Sie einige Sätze in Ihrer Antwort.

Does your paper address subitem 5-v?

Copy and paste relevant sections from the manuscript (include quotes in quotation marks "like this" to indicate direct quotes from your manuscript), or elaborate on this item by providing additional information not in the ms, or briefly explain why the item is not applicable/relevant for your study

The source code of the intervention cannot be specified because it is not owned by the German Depression Foundation, but by the european alliance against depression. The study design is described in sufficient detail for replication and the iFightDepression Tool can also be used in other research projects.

5-vi) Digital preservation

Digital preservation: Provide the URL of the application, but as the intervention is likely to change or disappear over the course of the years; also make sure the intervention is archived (Internet Archive, webcitation.org, and/or publishing the source code or screenshots/videos alongside the article). As pages behind login screens cannot be archived, consider creating demo pages which are accessible without login.

	1	2	3	4	5	
subitem not at all important	○	○	◉	○	○	essential

Does your paper address subitem 5-vi?

Copy and paste relevant sections from the manuscript (include quotes in quotation marks "like this" to indicate direct quotes from your manuscript), or elaborate on this item by providing additional information not in the ms, or briefly explain why the item is not applicable/relevant for your study

Meine Antwort

5-vii) Access

Access: Describe how participants accessed the application, in what setting/context, if they had to pay (or were paid) or not, whether they had to be a member of specific group. If known, describe how participants obtained “access to the platform and Internet” [1]. To ensure access for editors/reviewers/readers, consider to provide a “backdoor” login account or demo mode for reviewers/readers to explore the application (also important for archiving purposes, see vi).

	1	2	3	4	5	
subitem not at all important	○	◉	○	○	○	essential

Ihre Antwort ist zu lang. Kürzen Sie einige Sätze in Ihrer Antwort.

Does your paper address subitem 5-vii? *

Copy and paste relevant sections from the manuscript (include quotes in quotation marks "like this" to indicate direct quotes from your manuscript), or elaborate on this item by providing additional information not in the ms, or briefly explain why the item is not applicable/relevant for your study

Interventions were offered free of charge and no reimbursement was offered to participants.

5-viii) Mode of delivery, features/functionalities/components of the intervention and comparator, and the theoretical framework

Describe mode of delivery, features/functionalities/components of the intervention and comparator, and the theoretical framework [6] used to design them (instructional strategy [1], behaviour change techniques, persuasive features, etc., see e.g., [7, 8] for terminology). This includes an in-depth description of the content (including where it is coming from and who developed it) [1],” whether [and how] it is tailored to individual circumstances and allows users to track their progress and receive feedback” [6]. This also includes a description of communication delivery channels and – if computer-mediated communication is a component – whether communication was synchronous or asynchronous [6]. It also includes information on presentation strategies [1], including page design principles, average amount of text on pages, presence of hyperlinks to other resources, etc. [1].

	1	2	3	4	5	
subitem not at all important	○	○	○	◉	○	essential

Ihre Antwort ist zu lang. Kürzen Sie einige Sätze in Ihrer Antwort.

Does your paper address subitem 5-viii? *

Copy and paste relevant sections from the manuscript (include quotes in quotation marks "like this" to indicate direct quotes from your manuscript), or elaborate on this item by providing additional information not in the ms, or briefly explain why the item is not applicable/relevant for your study

"The iFightDepression® tool (iFD tool)
The iFightDepression® Tool is a guided online self-management tool, based on the principles of CBT. It includes six core workshops, each comprising written information, worksheets, exercises and a mood rating. For this study, participants were asked to use the tool for six weeks and to complete one workshop per week. Each week's workshop covered a different topic (e.g. an activity diary, monitoring and adapting one's sleep or challenging automated negative thoughts). The content and development is described in more details elsewhere (Oehler et al., 2019, Arensman et al., 2015). IFD offers the opportunity to complete worksheets online or to use a printed version. Patients were asked to try out each workshop and, if helpful, continue using the learned techniques. The intervention was not changed during the study period, except for a news box on the landing page that was updated approximately once a month.
Progressive muscle relaxation (PMR)
In the present study, PMR is used as the control condition. During the six weeks of intervention, participants are encouraged to practice PMR and learn how to deliberately induce physical relaxation to reduce stress and mental tension. Lessons range from 13-33 minutes and build on one another, adding more muscle groups every week. At the beginning of each week, participants receive a link to download the next lesson. They are instructed to practice on a daily basis, if possible, but at least two or three times a week and to integrate the practice into their daily routine." [...]
"The content and development is described in more details elsewhere (Oehler et al., 2019, Arensman et al., 2015)"

5-ix) Describe use parameters

Describe use parameters (e.g., intended "doses" and optimal timing for use). Clarify what instructions or recommendations were given to the user, e.g., regarding timing, frequency, heaviness of use, if any, or was the intervention used ad libitum.

	1	2	3	4	5	
subitem not at all important	○	○	○	○	◉	essential

Ihre Antwort ist zu lang. Kürzen Sie einige Sätze in Ihrer Antwort.

Does your paper address subitem 5-ix?

Copy and paste relevant sections from the manuscript (include quotes in quotation marks "like this" to indicate direct quotes from your manuscript), or elaborate on this item by providing additional information not in the ms, or briefly explain why the item is not applicable/relevant for your study

"The completion of the intervention was assessed each week using a self-report measure with two items. Participants were asked how often they had worked with the intervention during the last week and how much time they had spent on it.
Tracking the objective usage was possible only for the iFightDepression® group through the log-files of the iFightDepression® website. Offline use could not be tracked. For the PMR group, we tracked if participants downloaded the weekly changing intervention files. The actual use could not be tracked. Due to these limitations, both objective measures only served as an approximation and validation of the subjective measures."

5-x) Clarify the level of human involvement

Clarify the level of human involvement (care providers or health professionals, also technical assistance) in the e-intervention or as co-intervention (detail number and expertise of professionals involved, if any, as well as "type of assistance offered, the timing and frequency of the support, how it is initiated, and the medium by which the assistance is delivered". It may be necessary to distinguish between the level of human involvement required for the trial, and the level of human involvement required for a routine application outside of a RCT setting (discuss under item 21 – generalizability).

	1	2	3	4	5	
subitem not at all important	○	○	○	◉	○	essential

Ihre Antwort ist zu lang. Kürzen Sie einige Sätze in Ihrer Antwort.

Does your paper address subitem 5-x?

Copy and paste relevant sections from the manuscript (include quotes in quotation marks "like this" to indicate direct quotes from your manuscript), or elaborate on this item by providing additional information not in the ms, or briefly explain why the item is not applicable/relevant for your study

"Documentation of Guidance
The duration of every call made by the study assistants was recorded and added up to provide a sum score for the overall guidance received by each patient. Additionally, the content and perceived quality was rated by the study assistants. During the telephone calls guidance was provided according to a protocol developed for this purpose according to the guidelines for physicians and therapists using the iFightDepression® tool. Adverse events were recorded and topics relevant to the study, e.g. date of next appointment, were discussed." [...]
"Guidance
Guidance was provided during five telephone calls by the study assistants (psychologists and psychotherapists) from the Research Centre of the German Depression Foundation, supervised by a senior psychiatrist who was involved in the development of the iFD tool. Comparable to iFD guides outside of the study setting, all study assistants qualified using the standard webinar and used a guideline for the calls based on the webinar content. The focus of the guidance calls was on motivating the participants, rather than discussing the intervention content.
To keep contact with the study staff comparable across both intervention groups, the same guideline was used in the guidance calls for both the iFD and the PMR group and calls were carried out by the same study assistants."

5-xi) Report any prompts/reminders used

Report any prompts/reminders used: Clarify if there were prompts (letters, emails, phone calls, SMS) to use the application, what triggered them, frequency etc. It may be necessary to distinguish between the level of prompts/reminders required for the trial, and the level of prompts/reminders for a routine application outside of a RCT setting (discuss under item 21 – generalizability).

	1	2	3	4	5	
subitem not at all important	○	○	○	◉	○	essential

Ihre Antwort ist zu lang. Kürzen Sie einige Sätze in Ihrer Antwort.

Does your paper address subitem 5-xi? *

Copy and paste relevant sections from the manuscript (include quotes in quotation marks "like this" to indicate direct quotes from your manuscript), or elaborate on this item by providing additional information not in the ms, or briefly explain why the item is not applicable/relevant for your study

"Guidance was provided during five telephone calls by the study assistants (psychologists and psychotherapists) from the Research Centre of the German Depression Foundation, supervised by a senior psychiatrist who was involved in the development of the iFD tool. Comparable to iFD guides outside of the study setting, all study assistants qualified using the standard webinar and used a guideline for the calls based on the webinar content. The focus of the guidance calls was on motivating the participants, rather than discussing the intervention content." Further details can be found in the published study protocol.

5-xii) Describe any co-interventions (incl. training/support)

Describe any co-interventions (incl. training/support): Clearly state any interventions that are provided in addition to the targeted eHealth intervention, as ehealth intervention may not be designed as stand-alone intervention. This includes training sessions and support [1]. It may be necessary to distinguish between the level of training required for the trial, and the level of training for a routine application outside of a RCT setting (discuss under item 21 – generalizability.

	1	2	3	4	5	
subitem not at all important	○	◉	○	○	○	essential

Does your paper address subitem 5-xii? *

Copy and paste relevant sections from the manuscript (include quotes in quotation marks "like this" to indicate direct quotes from your manuscript), or elaborate on this item by providing additional information not in the ms, or briefly explain why the item is not applicable/relevant for your study

No additional co-interventions were offered, with the exception of the for the guidance as described above.

6a) Completely defined pre-specified primary and secondary outcome measures, including how and when they were assessed

Ihre Antwort ist zu lang. Kürzen Sie einige Sätze in Ihrer Antwort.

Does your paper address CONSORT subitem 6a? *

Copy and paste relevant sections from the manuscript (include quotes in quotation marks "like this" to indicate direct quotes from your manuscript), or elaborate on this item by providing additional information not in the ms, or briefly explain why the item is not applicable/relevant for your study

"Primary outcome
The Inventory of Depressive Symptomatology in its German self-report version (IDS-SR) was used as the primary outcome measure in this study (range: 0-84). The questionnaire was administered three times (see figure 2) to measure changes in depression severity. The scale has been shown to be useful in detecting symptom change as well as residual symptoms in depressed patients [23]. The concordant validity with the Beck Depression Inventory and the Hamilton Rating Scale for Depression has been shown to be appropriate (r ≥.88) [24].
Secondary outcomes
To assess changes in the perceived health-related quality of life, the Short-Form 12 (SF-12) scale was used. It was developed as a practical short form of the SF-36. A mental and a physical component score (both ranging from 0-100) can be calculated from the questionnaire answers for which moderate to high convergent validity has been shown in several studies [25,26].
The German version (ZUF-8) of the client satisfaction questionnaire (CSQ-8) was used to assess acceptance and feasibility of the interventions. Since the questionnaire was originally developed for the evaluation of hospital stays, the wording was slightly adapted to fit web-based interventions. A similar adaption yielded a good internal consistency (omega = .95) [27]."

6a-i) Online questionnaires: describe if they were validated for online use and apply CHERRIES items to describe how the questionnaires were designed/deployed

If outcomes were obtained through online questionnaires, describe if they were validated for online use and apply CHERRIES items to describe how the questionnaires were designed/deployed [9].

	1	2	3	4	5	
subitem not at all important	○	○	●	○	○	essential

Ihre Antwort ist zu lang. Kürzen Sie einige Sätze in Ihrer Antwort.

Does your paper address subitem 6a-i?

Copy and paste relevant sections from manuscript text

"During the six-week intervention period, the participants were asked to complete online questionnaires weekly (for details see figure 2). " More details on the deployment of the online questionnaires are given in the study protocol.

6a-ii) Describe whether and how "use" (including intensity of use/dosage) was defined/measured/monitored

Describe whether and how "use" (including intensity of use/dosage) was defined/measured/monitored (logins, logfile analysis, etc.). Use/adoption metrics are important process outcomes that should be reported in any ehealth trial.

	1	2	3	4	5	
subitem not at all important	○	○	○	○	◉	essential

Does your paper address subitem 6a-ii?

Copy and paste relevant sections from manuscript text

"The completion of the intervention was assessed each week using a self-report measure with two items. Participants were asked how often they had worked with the intervention during the last week and how much time they had spent on it.

Tracking the objective usage was possible only for the iFightDepression® group through the log-files of the iFightDepression® website. Offline use could not be tracked. For the PMR group, we tracked if participants downloaded the weekly changing intervention files. The actual use could not be tracked. Due to these limitations, both objective measures only served as an approximation and validation of the subjective measures."

6a-iii) Describe whether, how, and when qualitative feedback from participants was obtained

Describe whether, how, and when qualitative feedback from participants was obtained (e.g., through emails, feedback forms, interviews, focus groups).

	1	2	3	4	5	
subitem not at all important	○	○	○	◉	○	essential

Ihre Antwort ist zu lang. Kürzen Sie einige Sätze in Ihrer Antwort.

Does your paper address subitem 6a-iii?

Copy and paste relevant sections from manuscript text

This item does not apply to the current publication. to answer this research question no qualitative data analysis has been performe

6b) Any changes to trial outcomes after the trial commenced, with reasons

Does your paper address CONSORT subitem 6b? *

Copy and paste relevant sections from the manuscript (include quotes in quotation marks "like this" to indicate direct quotes from your manuscript), or elaborate on this item by providing additional information not in the ms, or briefly explain why the item is not applicable/relevant for your study

The trial outcomes have not been changed after the trial commenced.

7a) How sample size was determined

NPT: When applicable, details of whether and how the clustering by care provides or centers was addressed

7a-i) Describe whether and how expected attrition was taken into account when calculating the sample size

Describe whether and how expected attrition was taken into account when calculating the sample size.

	1	2	3	4	5	
subitem not at all important	○	○	○	◉	○	essential

Ihre Antwort ist zu lang. Kürzen Sie einige Sätze in Ihrer Antwort.

Does your paper address subitem 7a-i?

Copy and paste relevant sections from manuscript title (include quotes in quotation marks "like this" to indicate direct quotes from your manuscript), or elaborate on this item by providing additional information not in the ms, or briefly explain why the item is not applicable/relevant for your study

"As, to our best knowledge, this is the first RCT comparing guided online self-management to relaxation training as a credible active control, the magnitude of the expected effect could be estimated only approximately. Based on the results of a study conducted by Hegerl et al. [22], which compared face-to-face CBT to an active control condition (guided self-help group) and found a difference of 5.3 points on the Inventory of Depressive Symptomatology (IDS-C) after ten weeks, we estimated the difference in the present trial to be 4.0 points on the IDS-SR. To detect a difference of 4.0 points on the IDS with a power of 80% (alpha level = .05), the number of cases needed per group is 122. It was planned to include 360 participants to obtain at least 250 data sets after an expected drop-out of about 30%."

7b) When applicable, explanation of any interim analyses and stopping guidelines

Does your paper address CONSORT subitem 7b? *

Copy and paste relevant sections from the manuscript (include quotes in quotation marks "like this" to indicate direct quotes from your manuscript), or elaborate on this item by providing additional information not in the ms, or briefly explain why the item is not applicable/relevant for your study

No interim analyses were planned or performed in the current trial.

8a) Method used to generate the random allocation sequence

NPT: When applicable, how care providers were allocated to each trial group

Does your paper address CONSORT subitem 8a? *

Copy and paste relevant sections from the manuscript (include quotes in quotation marks "like this" to indicate direct quotes from your manuscript), or elaborate on this item by providing additional information not in the ms, or briefly explain why the item is not applicable/relevant for your study

"All participants, who provided written informed consent and matched the in- and exclusion criteria were randomized using the minimization algorithm by Pocock [21], stratified for gender (male/female), depression severity (mild/moderate according to PHQ-9), and CBT-experience (present/absent) with an 80% chance [illegible]

Ihre Antwort ist zu lang. Kürzen Sie einige Sätze in Ihrer Antwort.

8b) Type of randomisation; details of any restriction (such as blocking and block size)

Does your paper address CONSORT subitem 8b? *

Copy and paste relevant sections from the manuscript (include quotes in quotation marks "like this" to indicate direct quotes from your manuscript), or elaborate on this item by providing additional information not in the ms, or briefly explain why the item is not applicable/relevant for your study

please see answer to 8a)

9) Mechanism used to implement the random allocation sequence (such as sequentially numbered containers), describing any steps taken to conceal the sequence until interventions were assigned

Does your paper address CONSORT subitem 9? *

Copy and paste relevant sections from the manuscript (include quotes in quotation marks "like this" to indicate direct quotes from your manuscript), or elaborate on this item by providing additional information not in the ms, or briefly explain why the item is not applicable/relevant for your study

The randomisation was conducted by an external data analysis center (details are described in the study protocol (see Oehler et al. 2019).
Both, the participants and the study assistants were informed about the allocation only after the T0 questionnaire was filled in.

10) Who generated the random allocation sequence, who enrolled participants, and who assigned participants to interventions

Does your paper address CONSORT subitem 10? *

Copy and paste relevant sections from the manuscript (include quotes in quotation marks "like this" to indicate direct quotes from your manuscript), or elaborate on this item by providing additional information not in the ms, or briefly explain why the item is not applicable/relevant for your study

Participant enrollment was carried out by the study assistants after the successfull screenings and when the signed consent form was optained
The randomisation was then carried out by the center for clinical studies of the university Leipzig each frieday for the participants enrolled during the past week.
Both, study assistans as well as participants were informed about

Ihre Antwort ist zu lang. Kürzen Sie einige Sätze in Ihrer Antwort.

11a) If done, who was blinded after assignment to interventions (for example, participants, care providers, those assessing outcomes) and how

NPT: Whether or not administering co-interventions were blinded to group assignment

11a-i) Specify who was blinded, and who wasn’t

Specify who was blinded, and who wasn’t. Usually, in web-based trials it is not possible to blind the participants [1, 3] (this should be clearly acknowledged), but it may be possible to blind outcome assessors, those doing data analysis or those administering co-interventions (if any).

	1	2	3	4	5	
subitem not at all important	○	○	○	○	◉	essential

Does your paper address subitem 11a-i? *

Copy and paste relevant sections from the manuscript (include quotes in quotation marks "like this" to indicate direct quotes from your manuscript), or elaborate on this item by providing additional information not in the ms, or briefly explain why the item is not applicable/relevant for your study

After the allocation to one treatment option blinding was not possible for participants or study assistants providing guidance. The outcomes of the current study are self-report measures, so no blinded raters were involved.

11a-ii) Discuss e.g., whether participants knew which intervention was the “intervention of interest” and which one was the “comparator”

Informed consent procedures (4a-ii) can create biases and certain expectations - discuss e.g., whether participants knew which intervention was the “intervention of interest” and which one was the “comparator”.

	1	2	3	4	5	
subitem not at all important	○	○	○	○	◉	essential

Ihre Antwort ist zu lang. Kürzen Sie einige Sätze in Ihrer Antwort.

Does your paper address subitem 11a-ii?

Copy and paste relevant sections from the manuscript (include quotes in quotation marks "like this" to indicate direct quotes from your manuscript), or elaborate on this item by providing additional information not in the ms, or briefly explain why the item is not applicable/relevant for your study

"Individuals interested in taking part in the study were directed to a website providing general information on the study procedures and an online questionnaire assessing several in- and exclusion criteria. In the study information for participants both interventions were described as equivalent offers to not induce a bias through different expectations about the helpfullnes."

11b) If relevant, description of the similarity of interventions

(this item is usually not relevant for ehealth trials as it refers to similarity of a placebo or sham intervention to a active medication/intervention)

Does your paper address CONSORT subitem 11b? *

Copy and paste relevant sections from the manuscript (include quotes in quotation marks "like this" to indicate direct quotes from your manuscript), or elaborate on this item by providing additional information not in the ms, or briefly explain why the item is not applicable/relevant for your study

" To keep contact with the study staff comparable across both intervention groups, the same guideline was used in the guidance calls for both the iFD and the PMR group and calls were carried out by the same study assistants. "

"Progressive muscle relaxation (PMR)
In the present study, PMR is used as the control condition. [...]
It was chosen as a credible control intervention, being widely used in therapeutic settings, e.g. as part of cognitive behavioral therapy or in the treatment of sleep disorders. The method is also highly accepted by the public as a form of self-help for depression [31,32] as well as rated to be helpful by clinically depressed patients (38% very/moderately effective, 40% slightly effective [33]).
In a systematic review on several relaxation techniques (PMR or similar methods), relaxation was recommended as first line treatment in a stepped care approach. Antidepressant effects where visible shortly after relaxation interventions, superior to wait list and no treatment but inferior to psychotherapy [34], making PMR a suitable choice as a control condition that should be inferior to the web-based intervention."

Ihre Antwort ist zu lang. Kürzen Sie einige Sätze in Ihrer Antwort.

12a) Statistical methods used to compare groups for primary and secondary outcomes

NPT: When applicable, details of whether and how the clustering by care providers or centers was addressed

Does your paper address CONSORT subitem 12a? *

Copy and paste relevant sections from the manuscript (include quotes in quotation marks "like this" to indicate direct quotes from your manuscript), or elaborate on this item by providing additional information not in the ms, or briefly explain why the item is not applicable/relevant for your study

"To investigate changes over time in the primary and secondary outcomes, mixed model analyses were performed for each outcome measure including a random intercept and random slope for each participant, the variance-covariance structure was set to unstructured to not impose any constraints. This approach was taken to make the best use of incomplete data while minimizing the bias to the parameter estimations [36,37]. All analyses were performed on the intent-to-treat sample, using data from all randomized participants. As a sensitivity check, we repeated the analysis of the main outcome for a per-protocol sample (only participant having finished at least four workshops in the iFD tool or downloaded four sessions of PMR)." (answer shortened due to limited space)

12a-i) Imputation techniques to deal with attrition / missing values

Imputation techniques to deal with attrition / missing values: Not all participants will use the intervention/comparator as intended and attrition is typically high in ehealth trials. Specify how participants who did not use the application or dropped out from the trial were treated in the statistical analysis (a complete case analysis is strongly discouraged, and simple imputation techniques such as LOCF may also be problematic [4]).

	1	2	3	4	5	
subitem not at all important	○	○	○	○	◉	essential

Does your paper address subitem 12a-i? *

Copy and paste relevant sections from the manuscript (include quotes in quotation marks "like this" to indicate direct quotes from your manuscript), or elaborate on this item by providing additional information not in the ms, or briefly explain why the item is not applicable/relevant for your study

please see answer to item 12a)

Ihre Antwort ist zu lang. Kürzen Sie einige Sätze in Ihrer Antwort.

12b) Methods for additional analyses, such as subgroup analyses and adjusted analyses

Does your paper address CONSORT subitem 12b? *

Copy and paste relevant sections from the manuscript (include quotes in quotation marks "like this" to indicate direct quotes from your manuscript), or elaborate on this item by providing additional information not in the ms, or briefly explain why the item is not applicable/relevant for your study

please see answer to item 12a)

X26) REB/IRB Approval and Ethical Considerations [recommended as subheading under "Methods"] (not a CONSORT item)

X26-i) Comment on ethics committee approval

	1	2	3	4	5	
subitem not at all important	○	○	○	●	○	essential

Does your paper address subitem X26-i?

Copy and paste relevant sections from the manuscript (include quotes in quotation marks "like this" to indicate direct quotes from your manuscript), or elaborate on this item by providing additional information not in the ms, or briefly explain why the item is not applicable/relevant for your study

"The protocol for the current study was reviewed and approved by the ethics committee of the Medical Faculty, University of Leipzig on 2015-02-11."

x26-ii) Outline informed consent procedures

Outline informed consent procedures e.g., if consent was obtained offline or online (how? Checkbox, etc.?), and what information was provided (see 4a-ii). See [6] for some items to be included in informed consent documents.

	1	2	3	4	5	
subitem not at all important	○	○	○	●	○	essential

Ihre Antwort ist zu lang. Kürzen Sie einige Sätze in Ihrer Antwort.

Does your paper address subitem X26-ii?

Copy and paste relevant sections from the manuscript (include quotes in quotation marks "like this" to indicate direct quotes from your manuscript), or elaborate on this item by providing additional information not in the ms, or briefly explain why the item is not applicable/relevant for your study

"After successfully passing the questionnaire, contact details could be left for the main screening that took place via telephone. This procedure leads to a preselection of individuals with sufficient internet literacy to meet the minimal study requirements. All screening procedures and the guidance during the trial were carried out by psychologists or psychotherapists. If the screening was successful, participants were asked to provide written informed consent for participating in the study and to provide the telephone number of a confidant, whom the study team could contact in a suspected crisis (for further details on the screening and inclusion procedures see [19])."

X26-iii) Safety and security procedures

Safety and security procedures, incl. privacy considerations, and any steps taken to reduce the likelihood or detection of harm (e.g., education and training, availability of a hotline)

	1	2	3	4	5	
subitem not at all important	○	○	○	◉	○	essential

Does your paper address subitem X26-iii?

Copy and paste relevant sections from the manuscript (include quotes in quotation marks "like this" to indicate direct quotes from your manuscript), or elaborate on this item by providing additional information not in the ms, or briefly explain why the item is not applicable/relevant for your study

"The PHQ-9, a brief screening instrument, was filled out weekly by the participants and submitted to the study assistants for monitoring purposes. Patients reporting acute suicidality once or symptoms indicating severe depression for three weeks in a row, were contacted by the study assistants via telephone or email and, if necessary, advised to seek appropriate clinical support. A protocol on how to handle acute suicidality was established. Adverse events were coded at three time points (see figure 2) by the study assistants. Severity and possible associations to the study interventions were rated. Severe adverse events were passed on to the supervising physician for review. "

RESULTS

Ihre Antwort ist zu lang. Kürzen Sie einige Sätze in Ihrer Antwort.

13a) For each group, the numbers of participants who were randomly assigned, received intended treatment, and were analysed for the primary outcome

NPT: The number of care providers or centers performing the intervention in each group and the number of patients treated by each care provider in each center

Does your paper address CONSORT subitem 13a? *

Copy and paste relevant sections from the manuscript (include quotes in quotation marks "like this" to indicate direct quotes from your manuscript), or elaborate on this item by providing additional information not in the ms, or briefly explain why the item is not applicable/relevant for your study

Information on participant numbers are provided in detail in the participant flow chart (figure 1).
Of 496 participants, that were screened, 348 were included in the study and randomized (174 in the control condition and 173 in the intervention).
The main outcome measure was completed by 135 participants in the ifightdepression group and 129 participants in the control condition.

13b) For each group, losses and exclusions after randomisation, together with reasons

Does your paper address CONSORT subitem 13b? (NOTE: Preferably, this is shown in a CONSORT flow diagram) *

Copy and paste relevant sections from the manuscript (include quotes in quotation marks "like this" to indicate direct quotes from your manuscript), or elaborate on this item by providing additional information not in the ms, or briefly explain why the item is not applicable/relevant for your study

Drop-out,
n = 78
Reasons:
Other illness requiring treatment: n = 2
Non-compliance: n = 14
Worsening of depression: n = 5
Other : n = 57

Ihre Antwort ist zu lang. Kürzen Sie einige Sätze in Ihrer Antwort.

13b-i) Attrition diagram

Strongly recommended: An attrition diagram (e.g., proportion of participants still logging in or using the intervention/comparator in each group plotted over time, similar to a survival curve) or other figures or tables demonstrating usage/dose/engagement.

	1	2	3	4	5	
subitem not at all important	○	○	○	◉	○	essential

Does your paper address subitem 13b-i?

Copy and paste relevant sections from the manuscript or cite the figure number if applicable (include quotes in quotation marks "like this" to indicate direct quotes from your manuscript), or elaborate on this item by providing additional information not in the ms, or briefly explain why the item is not applicable/relevant for your study

While adherence and usage are described in the textbody of the manuscript, no diagram has been included. This is mainly due to the problem of not having an objective measure that is comparable across both groups.
The following information is given in the manuscript: "Out of the 348 included patients, 288 filled out the full T1 measure (after 3 weeks) and 262 completed T2 (after 6 weeks). Some of the measures reported on have deviating sample sizes (n) rates due to small number of participants omitting one or more of the measures."
For more details on usage please see the answer to Item 17a i)

14a) Dates defining the periods of recruitment and follow-up

Does your paper address CONSORT subitem 14a? *

Copy and paste relevant sections from the manuscript (include quotes in quotation marks "like this" to indicate direct quotes from your manuscript), or elaborate on this item by providing additional information not in the ms, or briefly explain why the item is not applicable/relevant for your study

"Recruitment for the trial commenced in June 2016 and was completed in August 2018 (follow-up data is still being collected and will be completed in August 2019). "

Ihre Antwort ist zu lang. Kürzen Sie einige Sätze in Ihrer Antwort.

14a-i) Indicate if critical “secular events” fell into the study period

Indicate if critical “secular events” fell into the study period, e.g., significant changes in Internet resources available or “changes in computer hardware or Internet delivery resources”

	1	2	3	4	5	
subitem not at all important	○	○	◉	○	○	essential

Does your paper address subitem 14a-i?

Copy and paste relevant sections from the manuscript (include quotes in quotation marks "like this" to indicate direct quotes from your manuscript), or elaborate on this item by providing additional information not in the ms, or briefly explain why the item is not applicable/relevant for your study

To our knowledge, no relevant secular events fell into the study period.

14b) Why the trial ended or was stopped (early)

Does your paper address CONSORT subitem 14b? *

Copy and paste relevant sections from the manuscript (include quotes in quotation marks "like this" to indicate direct quotes from your manuscript), or elaborate on this item by providing additional information not in the ms, or briefly explain why the item is not applicable/relevant for your study

The trial ended after the target sample size was achieved.

15) A table showing baseline demographic and clinical characteristics for each group

NPT: When applicable, a description of care providers (case volume, qualification, expertise, etc.) and centers (volume) in each group

Ihre Antwort ist zu lang. Kürzen Sie einige Sätze in Ihrer Antwort.

Does your paper address CONSORT subitem 15? *

Copy and paste relevant sections from the manuscript (include quotes in quotation marks "like this" to indicate direct quotes from your manuscript), or elaborate on this item by providing additional information not in the ms, or briefly explain why the item is not applicable/relevant for your study

"The participant flow chart (figure 1) provides an overview of the screening and enrollment numbers. Of the n = 347 patients, who were included into the study and randomized into one of the two treatment arms, 273 (78.7%) of these were female with a mean age of 42 (range 18-84, SD = 12.4). "
"According to self-reported medical histories, 321 (92.5%) of the participants had experienced at least one other episode of depression in the past (mean number of episodes: 14, range: 1-150, SD = 8.5) and had already tried several treatment options (for details see table 1). The most common comorbidities recorded using the M.I.N.I. were social phobia (48/347; 13.8%), agoraphobia/panic disorder (25/347; 7.2%) and generalized anxiety disorder (14/347; 4.0%)."
Please see table 1 for further baseline damographic and clinical characteristics for each group.

15-i) Report demographics associated with digital divide issues

In ehealth trials it is particularly important to report demographics associated with digital divide issues, such as age, education, gender, social-economic status, computer/Internet/ehealth literacy of the participants, if known.

	1	2	3	4	5	
subitem not at all important	○	○	○	◉	○	essential

Does your paper address subitem 15-i? *

Copy and paste relevant sections from the manuscript (include quotes in quotation marks "like this" to indicate direct quotes from your manuscript), or elaborate on this item by providing additional information not in the ms, or briefly explain why the item is not applicable/relevant for your study

"The level of education in the sample was high (218/347; 62.9% had graduated high school and, of these, 94/218; 43.1% had acquired a university degree); as was the internet literacy. Of our 347 participants, 312 (90%) had been using the internet for more than 10 years and 330 (95.1%) reported to have been using the internet on a daily basis."

16) For each group, number of participants (denominator) included in each analysis and whether the analysis was by

Ihre Antwort ist zu lang. Kürzen Sie einige Sätze in Ihrer Antwort.

16-i) Report multiple "denominators" and provide definitions

Report multiple "denominators" and provide definitions: Report N's (and effect sizes) "across a range of study participation [and use] thresholds" [1], e.g., N exposed, N consented, N used more than x times, N used more than y weeks, N participants "used" the intervention/comparator at specific pre-defined time points of interest (in absolute and relative numbers per group). Always clearly define "use" of the intervention.

	1	2	3	4	5	
subitem not at all important	○	○	○	◉	○	essential

Does your paper address subitem 16-i? *

Copy and paste relevant sections from the manuscript (include quotes in quotation marks "like this" to indicate direct quotes from your manuscript), or elaborate on this item by providing additional information not in the ms, or briefly explain why the item is not applicable/relevant for your study

"Out of the 348 included patients, 288 filled out the full T1 measure (after 3 weeks) and 262 completed T2 (after 6 weeks). Some of the measures reported on have deviating sample sizes (n) rates due to small number of participants omitting one or more of the measures. "
The denominators for each analysis are provided in the tables displaying the results (table 2, 3,4).
"As a sensitivity check, we repeated the analysis of the main outcome for a per-protocol sample (only participant having finished at least four workshops in the iFD tool or downloaded four sessions of PMR). "
"The results did not change when using a per-protocol sample and are therefore not reported separately."

16-ii) Primary analysis should be intent-to-treat

Primary analysis should be intent-to-treat, secondary analyses could include comparing only "users", with the appropriate caveats that this is no longer a randomized sample (see 18-i).

	1	2	3	4	5	
subitem not at all important	○	○	○	○	◉	essential

Does your paper address subitem 16-ii?

Copy and paste relevant sections from the manuscript (include quotes in quotation marks "like this" to indicate direct quotes from your manuscript), or elaborate on this item by providing additional information not in the ms, or briefly explain why the item is not applicable/relevant for your study

"All analyses were performed on the intent-to-treat sample, using data from all randomized participants. "

Ihre Antwort ist zu lang. Kürzen Sie einige Sätze in Ihrer Antwort.

17a) For each primary and secondary outcome, results for each group, and the estimated effect size and its precision (such as 95% confidence interval)

Does your paper address CONSORT subitem 17a? *

Copy and paste relevant sections from the manuscript (include quotes in quotation marks "like this" to indicate direct quotes from your manuscript), or elaborate on this item by providing additional information not in the ms, or briefly explain why the item is not applicable/relevant for your study

Raw values, effect sizes and confidence intervalls for the primary and secondary outcomes are given in table 3.

17a-i) Presentation of process outcomes such as metrics of use and intensity of use

In addition to primary/secondary (clinical) outcomes, the presentation of process outcomes such as metrics of use and intensity of use (dose, exposure) and their operational definitions is critical. This does not only refer to metrics of attrition (13-b) (often a binary variable), but also to more continuous exposure metrics such as "average session length". These must be accompanied by a technical description how a metric like a "session" is defined (e.g., timeout after idle time) [1] (report under item 6a).

	1	2	3	4	5	
subitem not at all important	○	○	○	◉	○	essential

Ihre Antwort ist zu lang. Kürzen Sie einige Sätze in Ihrer Antwort.

Does your paper address subitem 17a-i?

Copy and paste relevant sections from the manuscript (include quotes in quotation marks "like this" to indicate direct quotes from your manuscript), or elaborate on this item by providing additional information not in the ms, or briefly explain why the item is not applicable/relevant for your study

"According to self-report participants in the intervention group used the iFD tool 23.6 (SD= 12.6) times in the mean and participants in the PMR group practiced 22.4 (SD = 9.5) times over the course of six weeks. They reported to have spent an average of 6.2 (SD = 4.7) hours using the iFD program and an average of 6.5 (SD = 5.4) hours using PMR. Neither of these differences reached significance (t(303.26) = 0.606, P = .545 and t(334.91) = 0.620, P = .536, respectively). Objective data, taken from the back-end of the iFD tool and the download page of the PMR website, confirmed regular use and downloads of both interventions. The iFD users completed an average of 5.5 (SD = 2.1) workshops and spent a mean of 3.8 (SD = 3.0) hours using the tool online over the course of 18.7 sessions (the end of a session was defined by >30 min of idle time after the last click, time after the last click has not been included in average usage time). PMR users downloaded on average of 4.2 of the 6 relaxation lessons (n = 165, SD = 1.86; for 10 participants downloads had to be enabled differently due to technical problems and were not trackable)."

17b) For binary outcomes, presentation of both absolute and relative effect sizes is recommended

Does your paper address CONSORT subitem 17b? *

Copy and paste relevant sections from the manuscript (include quotes in quotation marks "like this" to indicate direct quotes from your manuscript), or elaborate on this item by providing additional information not in the ms, or briefly explain why the item is not applicable/relevant for your study

not applicable

18) Results of any other analyses performed, including subgroup analyses and adjusted analyses, distinguishing pre-specified from exploratory

Ihre Antwort ist zu lang. Kürzen Sie einige Sätze in Ihrer Antwort.

Does your paper address CONSORT subitem 18? *

Copy and paste relevant sections from the manuscript (include quotes in quotation marks "like this" to indicate direct quotes from your manuscript), or elaborate on this item by providing additional information not in the ms, or briefly explain why the item is not applicable/relevant for your study

(this part is shortened due to limited space)
"Covariate analysis
[...]
The final model included the original fixed effects of time and group as well as their interaction as predictors. In addition, fixed effects for sex and the amount of guidance received over the course of the intervention were added to the model. [...] The fixed effect for sex was statistically significant (t(332.3) = -2.33, P = 0.020) and described a level difference in the data with male participants reporting fewer symptoms over the whole intervention period (model parameter for the fixed effect: -2.50, SD = 1.04). The significant effect of the amount of guidance (t(348.6) = 2.59, P = 0.010) showed that higher overall IDS-SR scores were associated with a greater amount of contact with the study team (model parameter for the fixed effect: 0.06, SD = 0.02). [...] The final model yielded and conditional R^2 of 0.74 and a marginal R^2 of 0.08."

Quality of life
In the mixed model describing the changes in quality of life (mental component score) the significant interaction of group*time (t(279.8)= 2.005, P = .046)) reflects an intervention effect in favor of the iFightDepression® tool, indicating a greater improvement of the quality of live for iFD users compared to participants in the PMR group. The mental component score rose significantly (t(303.6)= 2.946, P = .003)) over time, indicating an improvement in the mental component of quality of life in both groups over the intervention period. [...]

User Satisfaction
The mean sum score of the CSQ-8, a measure of user satisfaction was at 25.31 in the iFD group and at 21.97 in the PMR group out of a possible 32, the difference being statistically significant (t(259)=5.8044, P <.01). [...]"

18-i) Subgroup analysis of comparing only users

A subgroup analysis of comparing only users is not uncommon in ehealth trials, but if done, it must be stressed that this is a self-selected sample and no longer an unbiased sample from a randomized trial (see 16-iii).

1 2 3 4 5

Ihre Antwort ist zu lang. Kürzen Sie einige Sätze in Ihrer Antwort.

Does your paper address subitem 18-i?

Copy and paste relevant sections from the manuscript (include quotes in quotation marks "like this" to indicate direct quotes from your manuscript), or elaborate on this item by providing additional information not in the ms, or briefly explain why the item is not applicable/relevant for your study

"The results did not change when using a per-protocol sample and are therefore not reported separately."

19) All important harms or unintended effects in each group

(for specific guidance see CONSORT for harms)

Does your paper address CONSORT subitem 19? *

Copy and paste relevant sections from the manuscript (include quotes in quotation marks "like this" to indicate direct quotes from your manuscript), or elaborate on this item by providing additional information not in the ms, or briefly explain why the item is not applicable/relevant for your study

"Adverse Events
During the course of the intervention, 159 adverse events and four serious adverse events were recorded. Three adverse events were rated as "possibly related" to the intervention, two in the iFightDepression® group and one in the PMR group. The "possibly related" adverse events were all deteriorations of the patient's mood. One patient in each group described the feeling of trying one more intervention that did not help as a contributing factor to the deterioration and one participant in the iFightDepression® group reported worse symptoms of depression after reducing her antidepressants without consultation of her physician, since she expected to get better by using iFightDepression®. Of the serious adverse events, two occurred in each intervention group and none were rated as related or possibly related to the intervention."

19-i) Include privacy breaches, technical problems

Include privacy breaches, technical problems. This does not only include physical "harm" to participants, but also incidents such as perceived or real privacy breaches [1], technical problems, and other unexpected/unintended incidents. "Unintended effects" also includes unintended positive effects [2].

	1	2	3	4	5	
subitem not at all important	○	○	○	◉	○	essential

Ihre Antwort ist zu lang. Kürzen Sie einige Sätze in Ihrer Antwort.

Does your paper address subitem 19-i?

Copy and paste relevant sections from the manuscript (include quotes in quotation marks "like this" to indicate direct quotes from your manuscript), or elaborate on this item by providing additional information not in the ms, or briefly explain why the item is not applicable/relevant for your study

"...for 10 participants downloads had to be enabled differently due to technical problems and were not trackable"
Besides that, technical problems or no privacy breaches occured.

19-ii) Include qualitative feedback from participants or observations from staff/researchers

Include qualitative feedback from participants or observations from staff/researchers, if available, on strengths and shortcomings of the application, especially if they point to unintended/unexpected effects or uses. This includes (if available) reasons for why people did or did not use the application as intended by the developers.

	1	2	3	4	5	
subitem not at all important	○	○	◉	○	○	essential

Does your paper address subitem 19-ii?

Copy and paste relevant sections from the manuscript (include quotes in quotation marks "like this" to indicate direct quotes from your manuscript), or elaborate on this item by providing additional information not in the ms, or briefly explain why the item is not applicable/relevant for your study

Meine Antwort

DISCUSSION

22) Interpretation consistent with results, balancing benefits and harms, and considering other relevant evidence

NPT: In addition, take into account the choice of the comparator, lack of or partial blinding, and unequal expertise of care providers or centers in each group

Ihre Antwort ist zu lang. Kürzen Sie einige Sätze in Ihrer Antwort.

22-i) Restate study questions and summarize the answers suggested by the data, starting with primary outcomes and process outcomes (use)

Restate study questions and summarize the answers suggested by the data, starting with primary outcomes and process outcomes (use).

	1	2	3	4	5	
subitem not at all important	○	○	○	○	◉	essential

Does your paper address subitem 22-i? *

Copy and paste relevant sections from the manuscript (include quotes in quotation marks "like this" to indicate direct quotes from your manuscript), or elaborate on this item by providing additional information not in the ms, or briefly explain why the item is not applicable/relevant for your study

"The current study is one of the first studies on antidepressant effects of iCBT using a control condition which is not likely to induce nocebo effects but is both credible and comparable to the iCBT group in terms of duration and contact with the study team. This setting allowed to control for unspecific treatment effects such as hope induction and activation that might be evoked through the study setting as such. To this end, we assessed between-group differences concerning changes in symptom severity over time as the primary outcome and differences in health-related quality of life as a secondary outcome.
The main outcome (decrease in symptoms of depression as measured with the IDS-SR) did not differ significantly between the intervention and the control group (small effect), yielding different results from Mackinnon et al. [15] and Johansson et al. [16], who observed small, but significant effects when comparing internet based interventions to active control conditions"

22-ii) Highlight unanswered new questions, suggest future research

Highlight unanswered new questions, suggest future research.

	1	2	3	4	5	
subitem not at all important	○	◉	○	○	○	essential

Ihre Antwort ist zu lang. Kürzen Sie einige Sätze in Ihrer Antwort.

Does your paper address subitem 22-ii?

Copy and paste relevant sections from the manuscript (include quotes in quotation marks "like this" to indicate direct quotes from your manuscript), or elaborate on this item by providing additional information not in the ms, or briefly explain why the item is not applicable/relevant for your study

Meine Antwort

20) Trial limitations, addressing sources of potential bias, imprecision, and, if relevant, multiplicity of analyses

20-i) Typical limitations in ehealth trials

Typical limitations in ehealth trials: Participants in ehealth trials are rarely blinded. Ehealth trials often look at a multiplicity of outcomes, increasing risk for a Type I error. Discuss biases due to non-use of the intervention/usability issues, biases through informed consent procedures, unexpected events.

	1	2	3	4	5	
subitem not at all important	○	○	○	○	◉	essential

Does your paper address subitem 20-i? *

Copy and paste relevant sections from the manuscript (include quotes in quotation marks "like this" to indicate direct quotes from your manuscript), or elaborate on this item by providing additional information not in the ms, or briefly explain why the item is not applicable/relevant for your study

The limitations of the study are described in a sepparate section of the discussion. Due to limited space in the questionaire, the section could not be copied in full. The possibility of the control condition not being a true placebo is discussed, as well as the differences in guidance and the probability, that the study was underpowered. Also, the specific characteristics of the current sample are discussed.

21) Generalisability (external validity, applicability) of the trial findings

NPT: External validity of the trial findings according to the intervention, comparators, patients, and care providers or centers involved in the trial

Ihre Antwort ist zu lang. Kürzen Sie einige Sätze in Ihrer Antwort.

21-i) Generalizability to other populations

Generalizability to other populations: In particular, discuss generalizability to a general Internet population, outside of a RCT setting, and general patient population, including applicability of the study results for other organizations

	1	2	3	4	5	
subitem not at all important	○	○	○	○	◉	essential

Does your paper address subitem 21-i?

Copy and paste relevant sections from the manuscript (include quotes in quotation marks "like this" to indicate direct quotes from your manuscript), or elaborate on this item by providing additional information not in the ms, or briefly explain why the item is not applicable/relevant for your study

"For further interpretation of the current results the composition of the sample should be considered: The current sample was a self-selected community sample with a high rate of very educated, internet affine patients who had experienced several episodes of depression in the past (mean = 14), who had already gained experience with psychotherapy (past: 330/347, 95,1%, present: 190/347; 54.8%) and who were receiving some kind of treatment at the beginning of the study. The mean age of the onset of depression was at 21.8 (SD = 12.1), indicating that, on average, participants had been experiencing episodes of depression for about 20 years before participating in this study. This differs widely from the treatment rates in the study by Johansson et al. [16], where 46.2% of the participants had never experienced psychological treatment and 38.5% had not been treated with medication.
This implicates that the participants in the current study could have already gained a lot of experience in therapeutic techniques and in managing their symptoms, which might have attenuated the treatment effects in comparison to studies treating patients with a first episode of depression or less treatment experience."

21-ii) Discuss if there were elements in the RCT that would be different in a routine application setting

Discuss if there were elements in the RCT that would be different in a routine application setting (e.g., prompts/reminders, more human involvement, training sessions or other co-interventions) and what impact the omission of these elements could have on use, adoption, or outcomes if the intervention is applied outside of a RCT setting.

	1	2	3	4	5	
subitem not at all important	○	○	○	○	◉	essential

Ihre Antwort ist zu lang. Kürzen Sie einige Sätze in Ihrer Antwort.

Does your paper address subitem 21-ii?

Copy and paste relevant sections from the manuscript (include quotes in quotation marks "like this" to indicate direct quotes from your manuscript), or elaborate on this item by providing additional information not in the ms, or briefly explain why the item is not applicable/relevant for your study

"In case of the IFD tool, the high usage rates with minimal guidance is good news, since it is supposed to be guided by therapists and physicians in routine care with limited time in their day-to-day work. Still, the highly structured study environment with an extensive screening interview (not counted as guidance time in this study) and regular questionnaires might have further improved adherence. This should be kept in mind, when implementing iFD in routine care."

OTHER INFORMATION

23) Registration number and name of trial registry

Does your paper address CONSORT subitem 23? *

Copy and paste relevant sections from the manuscript (include quotes in quotation marks "like this" to indicate direct quotes from your manuscript), or elaborate on this item by providing additional information not in the ms, or briefly explain why the item is not applicable/relevant for your study

"Trial registration: International Clinical Trials Registry Platform (ICTRP), ID 080-15-09032015. "

24) Where the full trial protocol can be accessed, if available

Does your paper address CONSORT subitem 24? *

Cite a Multimedia Appendix, other reference, or copy and paste relevant sections from the manuscript (include quotes in quotation marks "like this" to indicate direct quotes from your manuscript), or elaborate on this item by providing additional information not in the ms, or briefly explain why the item is not applicable/relevant for your study

Oehler C, Görges F, Böttger D et al. Efficacy of an internet-based self-management intervention for depression or dysthymia - a study protocol of an RCT using an active control condition. BMC Psychiatry. 2019 Mar 14;19(1):90. PMID:30871544

Ihre Antwort ist zu lang. Kürzen Sie einige Sätze in Ihrer Antwort.

25) Sources of funding and other support (such as supply of drugs), role of funders

Does your paper address CONSORT subitem 25? *

Copy and paste relevant sections from the manuscript (include quotes in quotation marks "like this" to indicate direct quotes from your manuscript), or elaborate on this item by providing additional information not in the ms, or briefly explain why the item is not applicable/relevant for your study

"The trial was funded within the framework of the cooperation between the German Depression Foundation and the 'Deutsche Bahn Stiftung gGmbH'."

X27) Conflicts of Interest (not a CONSORT item)

X27-i) State the relation of the study team towards the system being evaluated

In addition to the usual declaration of interests (financial or otherwise), also state the relation of the study team towards the system being evaluated, i.e., state if the authors/evaluators are distinct from or identical with the developers/sponsors of the intervention.

	1	2	3	4	5	
subitem not at all important	○	○	○	◉	○	essential

Does your paper address subitem X27-i?

Copy and paste relevant sections from the manuscript (include quotes in quotation marks "like this" to indicate direct quotes from your manuscript), or elaborate on this item by providing additional information not in the ms, or briefly explain why the item is not applicable/relevant for your study

UH is member of the advisory board for Janssen Pharmaceutica, received travel costs and honorarium as speaker, research grant for an investigator initiated trial from Medice and travel costs and honorarium as speaker for Servier.
"CRK received lecture honoraria by Servier.
UH, CRK, MR, FG and CO work or have worked for the German Depression Foundation, implementing the iFightDepression tool in Germany.
The authors declare that they have no further competing interests."

About the CONSORT EHEALTH checklist

Ihre Antwort ist zu lang. Kürzen Sie einige Sätze in Ihrer Antwort.

As a result of using this checklist, did you make changes in your manuscript? *

- ◯ yes, major changes
- ◉ yes, minor changes
- ◯ no

What were the most important changes you made as a result of using this checklist?

The checklist was helpfull to clarify minor points and add more details, mainly in the methods section.

How much time did you spend on going through the checklist INCLUDING making changes in your manuscript *

This was done over the course of several working days, so it is difficult to track the exact time. Filling in the checklist and making changes to the manuscript probably took <10 hours. Since the list can not be saved temporarily for later use, it was completed in a word document and it took time again to copy the answers to the online form.

As a result of using this checklist, do you think your manuscript has improved? *

- ◉ yes
- ◯ no
- ◯ Sonstiges:

Ihre Antwort ist zu lang. Kürzen Sie einige Sätze in Ihrer Antwort.

Would you like to become involved in the CONSORT EHEALTH group?

This would involve for example becoming involved in participating in a workshop and writing an "Explanation and Elaboration" document

- ○ yes
- ◉ no
- ○ Sonstiges:

Any other comments or questions on CONSORT EHEALTH

It would be extremely helpfull to be able to save the checklist and continue working with it the next time. Also, when I tried to submit the answers, I got a warning that some answers were to long and needed shortening, but the form did not indicate which answers needed changing.

STOP - Save this form as PDF before you click submit

To generate a record that you filled in this form, we recommend to generate a PDF of this page (on a Mac, simply select "print" and then select "print as PDF") before you submit it.

When you submit your (revised) paper to JMIR, please upload the PDF as supplementary file.

Don't worry if some text in the textboxes is cut off, as we still have the complete information in our database. Thank you!

Final step: Click submit !

Click submit so we have your answers in our database!

SENDEN

Geben Sie niemals Passwörter über Google Formulare weiter.

Ihre Antwort ist zu lang. Kürzen Sie einige Sätze in Ihrer Antwort.

Google Formulare

Ihre Antwort ist zu lang. Kürzen Sie einige Sätze in Ihrer Antwort.
